# Proteome Exploration of *Legionella pneumophila* To Identify Novel Therapeutics: a Hierarchical Subtractive Genomics and Reverse Vaccinology Approach

**DOI:** 10.1128/spectrum.00373-22

**Published:** 2022-07-12

**Authors:** Md Tahsin Khan, Araf Mahmud, Mahmudul Hasan, Kazi Faizul Azim, Musammat Kulsuma Begum, Mohimenul Haque Rolin, Arzuba Akter, Shakhinur Islam Mondal

**Affiliations:** a Department of Genetic Engineering and Biotechnology, Shahjalal University of Science and Technology, Sylhet, Bangladesh; b Department of Pharmaceuticals and Industrial Biotechnology, Sylhet Agricultural University, Sylhet, Bangladesh; c Department of Microbial Biotechnology, Sylhet Agricultural University, Sylhet, Bangladesh; d Department of Biochemistry and Molecular Biology, Shahjalal University of Science and Technology, Sylhet, Bangladesh; McGill University

**Keywords:** *Legionella pneumophila*, Legionnaires’ disease, molecular dynamics, protein-protein docking, reverse vaccinology, subtractive genomics

## Abstract

Legionella pneumophila is the causative agent of a severe type of pneumonia (lung infection) called Legionnaires’ disease. It is emerging as an antibiotic-resistant strain day by day. Hence, identifying novel drug targets and vaccine candidates is essential to fight against this pathogen. Here, attempts were taken through a subtractive genomics approach on the complete proteome of L. pneumophila to address the challenges of multidrug resistance. A total of 2,930 proteins from L. pneumophila proteome were investigated through diverse subtractive proteomics approaches, e.g., identification of human nonhomologous and pathogen-specific essential proteins, druggability and “anti-target” analysis, subcellular localization prediction, human microbiome nonhomology screening, and protein-protein interaction studies to find out effective drug and vaccine targets. Only three fulfilled these criteria and were proposed as novel drug targets against L. pneumophila. Furthermore, outer membrane protein TolB was identified as a potential vaccine target with a better antigenicity score. Antigenicity and transmembrane topology screening, allergenicity and toxicity assessment, population coverage analysis, and a molecular docking approach were adopted to generate the most potent epitopes. The final vaccine was constructed by the combination of highly immunogenic epitopes, along with suitable adjuvant and linkers. The designed vaccine construct showed higher binding interaction with different major histocompatibility complex (MHC) molecules and human immune TLR-2 receptors with minimum deformability at the molecular level. The present study aids the development of novel therapeutics and vaccine candidates for efficient treatment and prevention of L. pneumophila infections. However, further wet-lab-based phenotypic and genomic investigations and *in vivo* trials are highly recommended to validate our prediction experimentally.

**IMPORTANCE** Legionella pneumophila is a human pathogen distributed worldwide, causing Legionnaires’ disease (LD), a severe form of pneumonia and respiratory tract infection. L. pneumophila is emerging as an antibiotic-resistant strain, and controlling LD is now difficult. Hence, developing novel drugs and vaccines against L. pneumophila is a major research priority. Here, the complete proteome of L. pneumophila was considered for subtractive genomics approaches to address the challenge of antimicrobial resistance. Our subtractive proteomics approach identified three potential drug targets that are promising for future application. Furthermore, a possible vaccine candidate, “outer membrane protein TolB,” was proposed using reverse vaccinology analysis. The constructed vaccine candidate showed higher binding interaction with MHC molecules and human immune TLR-2 receptors at the molecular level. Overall, the present study aids in developing novel therapeutics and vaccine candidates for efficient treatment of the infections caused by L. pneumophila.

## INTRODUCTION

Legionella pneumophila is a human pathogen distributed worldwide within freshwater and biofilms (1–3). These fastidious Gram-negative aerobic bacilli belong to the family *Legionellaceae* and the genus *Legionella*. The genus includes 60 other species and, together, these species are divided into 80 serogroups (4). Inhalation of *Legionella* spp. causes Legionnaires’ disease (LD), a severe form of pneumonia and respiratory tract infection (5), while serogroup one alone is responsible for 70 to 90% of cases (6, 7). L. pneumophila is responsible for maximum incidences of water-associated diseases in the United States (8, 9) and caused more than half of all reported waterborne disease outbreaks in 2013 and 2014 (8), and the incidence rate of LD increased by 300% between 2005 and 2019 (10). It was declared an important pathogen by the U.S. Environmental Protection Agency (EPA) Contaminant Candidate List (CCL) due to its prevalence and the severity of its disease-causing effects (11). Diverse *Legionella* LD-causing species are genetically isolated from each other and selectively acquire genes with potential for enhanced virulence; however, they share a conserved core genome (12). Several virulence factors are encoded by distinct regions of DNA present in the pathogenicity island (PAI) of pathogenic bacteria (absent in nonpathogenic strains) (13–15). Moreover, *Legionella* possesses many of the traditional bacterial determinants that are important for pathogenicity in other bacteria, including pili, flagella, lipopolysaccharide (LPS), the type II secretion system, and several outer membrane proteins (OMPs), i.e., outer membrane vesicles, peptidoglycan-associated lipoprotein, major secreted phospholipase (PlaA), major cell-associated phospholipase A/lysophospholipase A (PlaB), major OMP, PlaC, macrophage infectivity potentiator, Hsp60, and FeoB (16). *Legionella* transmission from human to human generally does not occur or is rare and can only be acquired from environmental sources (17). The pathogen grows and localizes in hot, moist, and nutrient-rich sedimentary environments (18). A potable water distribution system was the culprit behind hospital-acquired Legionnaires’ disease (19).

Macrolides (azithromycin) and fluoroquinolones (levofloxacin, moxifloxacin, and ciprofloxacin) are the standard treatment for *Legionella* infection (20, 21). Current American and European guidelines recommend azithromycin and levofloxacin as the first-line treatment (22). A combination of fluoroquinolone and azithromycin is recommended in the case of drug resistance or severe LD pneumonia (23). The emergence of resistance against antibiotics or a combination of antibiotics for various infectious agents poses global threats in the human health field (24). Recent epidemiological research found that L. pneumophila strains display a high prevalence of tolerance (50 to 100%) to widely used antibiotics such as azithromycin, ceftriaxone, rifampicin, tigecycline, ciprofloxacin, moxifloxacin, doxycycline, erythromycin, levofloxacin, and clarithromycin (25, 26). Despite the success of macrolides and fluoroquinolone, resistance developed against these drugs resulted in treatment failures (27, 28). Horizontal gene transfer is a driving force of acquiring antibiotic resistance genes, resulting in genome plasticity in bacteria (29). Natural transformation by competence plays a crucial role in horizontally acquired resistance in bacteria (30). Antibiotics of the fluoroquinolone family and genotoxic stress caused by UV radiation and other DNA-damaging agents induce competence genes in L. pneumophila resulting in rapid uptake and propagation of exogenous DNA (5, 31). Horizontal gene transfer by conjugative plasmids facilitates the incorporation of antibiotic resistance genes (32). Even transposons and bacteriophages are suitable for transferring antibiotic-resistant genes in pathogenic bacteria (33). L. pneumophila contains a type IV secretion system that makes it ideal for incorporating conjugative plasmids, leading to plasmid-mediated antibiotic resistance genes (30). LpeA, a subunit of the LpeAB efflux pump in L. pneumophila (homologous to AcrA in Escherichia coli), was attributed to macrolide resistance in the L. pneumophila pathogen (34). Researchers also detected point mutations or insertions/deletions in the ribosomal genes *rplD* and *rplV* that increased the MICs of macrolides (27). Mutations in *gyrA*, *gyrB*, *parC*, and *parE* are associated with fluoroquinolone resistance (35). Therefore, there is an urgent need to develop new therapeutic antibacterial agents directed toward novel targets. A disease-based approach of the traditional drug discovery method is expensive and time-consuming since significant time and devoted researchers are required to identify potential ligands. In recent years, computational aided drug targets discovery methods reduce time consumption by eliminating compounds in this process that have a limited chance of success (36–38). Among various computational methods and strategies, subtractive and comparative microbial genomics approaches efficiently identified potential targets in several human pathogens (39). The main theme of subtractive and comparative genomics approaches is to find targets (gene/protein) that are essential for the pathogen and possess no homology counterpart in the host (40). Along with the subtractive genomics approach, the reverse vaccinology strategy optimizes the prediction and development of novel drug and vaccine targets, especially for microorganisms that are difficult to grow in the laboratory, such as intracellular bacteria, including L. pneumophila. Although successful in many cases, conventional or live-attenuated vaccines are often associated with several obstacles (41). Such vaccines may take decades to develop while relying on adequate antigen expression from *in vitro* culture models. Microorganisms are sometimes difficult to cultivate and, in some instances, attenuate (42, 43). Moreover, attenuation may cause cross-contamination and produce undesirable adverse immune responses (44). Epitope-based vaccine prediction, on the contrary, could be a faster approach to targeting immunogenic protein through the entire bacterial or viral proteome (45–47). The reverse vaccinology approach usually identifies a protein vaccine candidate using defined features, such as protein subcellular localization, topology, adhesion/antigenicity probability, epitopes, and binding to major histocompatibility complex (MHC) class I and II molecules (48). This approach has been proven to prioritize and design vaccine targets against multiple pathogens (49–52).

In this study, we utilized an in-depth subtractive genomics and reverse vaccinology approach to identify novel therapeutic drug and vaccine targets in L. pneumophila subsp. *pneumophila* (strain Philadelphia 1/ATCC 33152/DSM 7513). We particularly considered the key essential or survival proteins of the pathogen, which are nonhomologous to the host as well as host microbiota. We screened for outer membrane proteins (OMPs) and B- or T-cell epitopes. At the same time, we performed the human microbiome nonhomologous analysis. The highest scoring OMPs and epitopes will facilitate future *in vitro* and *in vivo* tests to produce drugs and suitable vaccine against intracellular L. pneumophila infection.

## RESULTS

**FIG 1:**
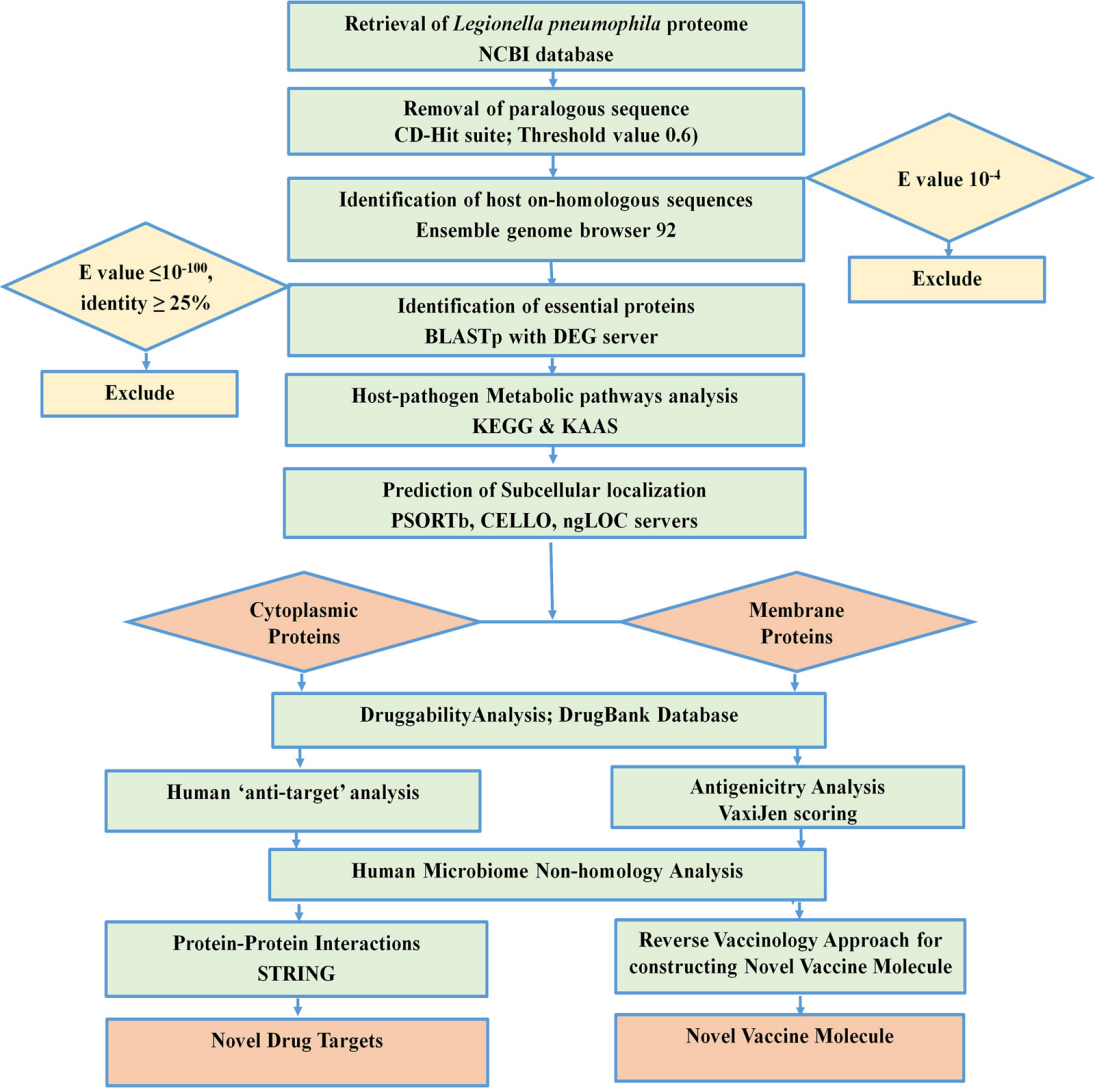
Proteome exploration of L. pneumophila to identify novel drug targets.

**FIG 2:**
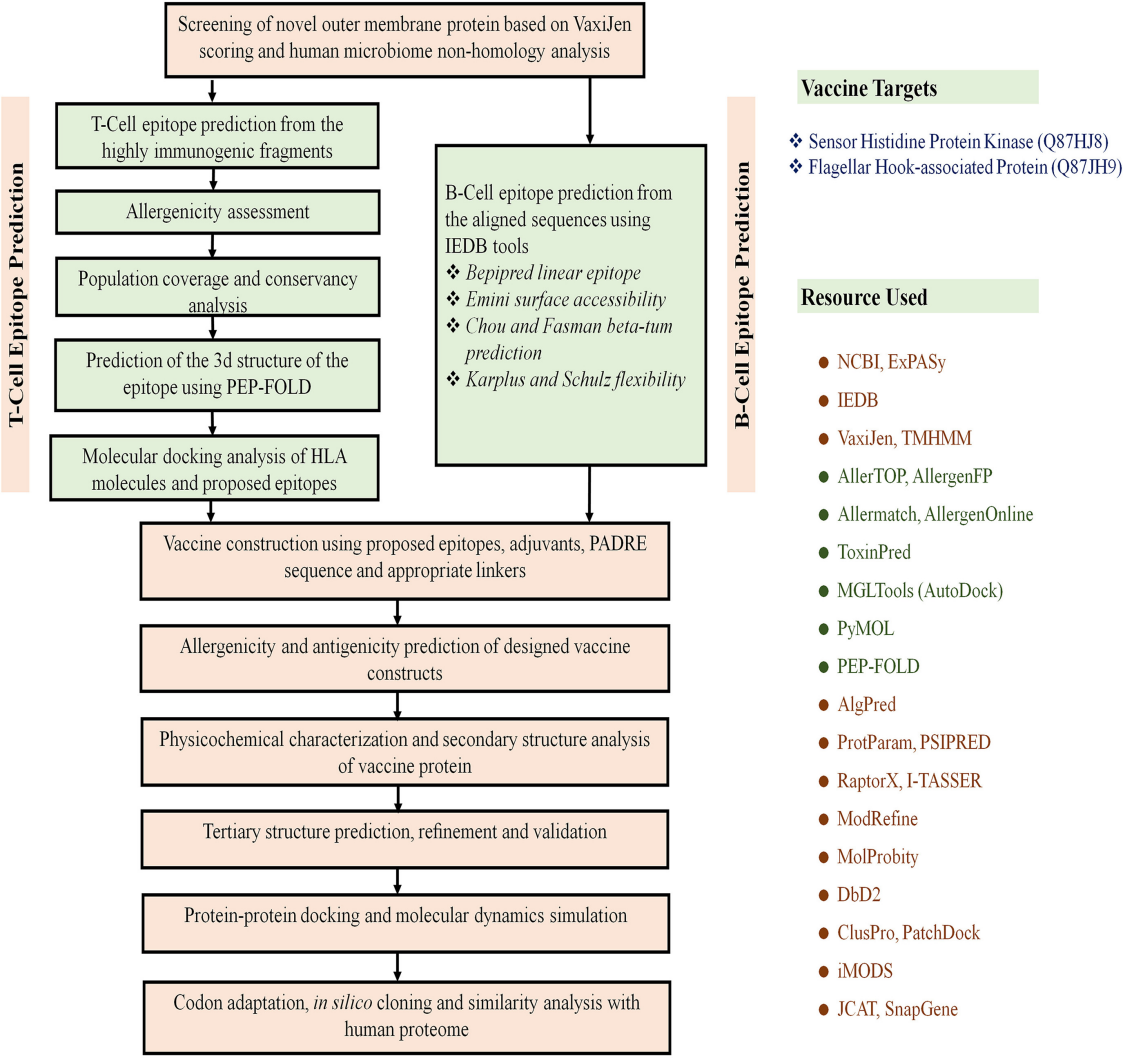
Flow chart summarizing the protocols for the prediction of epitope-based vaccine candidate by the *in silico* reverse vaccinology technique.

Genome-wide proteome exploration of L. pneumophila was employed to identify novel drug and vaccine targets through subtractive genomics and reverse vaccinology approaches. The subtractive genomics approach is a continuous process where irrelevant or nontarget sequences are allowed to be subtracted from a vast pool of initially retrieved sequences. Here, a list of human “anti-target” proteins was used to cross-check with the screened drug target alongside other common procedures of subtractive genomics. Moreover, the human microbiome was also checked with the screened putative drug candidates since the unintentional interference of proteins available in the gut microflora may cause adverse consequences on host physiology. After successfully screening sequences, we added reverse vaccinology approaches to get an insight into the potential design of vaccine candidates. During the entire procedure, the study utilized up-to-date bioinformatics approaches to discover novel therapeutics against L. pneumophila. The overall workflow has been illustrated in Fig. 1 and 2. All of the methodologies used in the proteome exploration of L. pneumophila, including the number of proteins screened in each step, are summarized in Table 1.

**TABLE 1:** Subtractive genomic analysis scheme toward the identification of novel therapeutic targets

Serial no.	Steps/description	L. pneumophila subsp. *pneumophila*^*a*^
1	Total no. of proteins	2930
2	No. of nonparalogous proteins (>60% identical) in CD-Hit	2878
3	No. of proteins nonhomologous to H. sapiens using BLASTp (E = 10^−3^)	2010
4	Essential proteins in DEG 15.2 server (E ≤ 10^−100^, bit score >100)	125
5	Essential proteins involved only in unique metabolic pathways (KAAS at KEGG)	4
6	Proteins assigned KO (KEGG orthology) but not assigned in any pathway	29
7	Hypothetical essential proteins assigned KO (KEGG orthology) but not assigned in any pathway	3
8	Essential membrane proteins using PSORTb, CELLO, ngLOC, PSLpred servers	12
9	Essential cytoplasmic proteins using PSORTb, CELLO, ngLOC, PSLpred servers	13
	Protein of unknown location	1
10	Essential proteins found to be novel in DrugBank 5.1.0	25
11	Proteins nonhomologous to “anti-targets” using BLASTp (E < 0.005, identity <30%, query length >30%)	25
12	Membrane proteins showing antigenicity using VaxiJen v2.0 (threshold value >0.4)	12
13	No. of cytoplasmic and membrane proteins less similar to human microbiota using BLASTp against “43021[BioProject]” (E = 1, identity <47%)	5
14	Proteins selected for epitope design	1
15	Novel drug targets	3

a Strain Philadelphia 1/ATCC 33152/DSM 7513.

### Proteome retrieval and finding paralogous sequences.

The whole proteome retrieved from UniProt (Proteome ID UP000000609) containing 2,930 proteins was analyzed in the CD-Hit tool using the threshold value 0.6 to eliminate paralogous and duplicate sequences showing >60% sequence identity. Paralogous protein sequences were removed, leaving 2,878 nonparalogous protein sequences.

### Identification of human nonhomologous proteins.

BLASTp screened nonparalogous protein sequences through Ensembl genome browser 92. A total of 868 proteins were found to be similar to the human genome, which was excluded, leaving a total of 2,010 nonhomologous proteins.

### Identification of essential proteins.

Among the 2,010 nonhomologous proteins, only 125 protein sequences showed similarity in sequence identity with the essential proteins enlisted in the Database of Essential Genes (DEG) server from 48 bacterial strains. These 125 proteins are listed as essential proteins and are responsible for the survival of L. pneumophila.

### Metabolic pathway analysis and family prediction of hypothetical proteins.

Although 120 pathways of target L. pneumophila were identified from the KEGG server, only 37 were detected as unique for the pathogen (see Table S1 in the supplemental material). The result from the KAAS server at KEGG revealed that 122 proteins among 125 had assigned KEGG Orthology (KO) identifiers. Of the 122 proteins, only 4 were involved in unique metabolic pathways (Table 2), and 29 were involved in neither unique nor common pathways (see Table S2). Only three hypothetical proteins were found among these 29 KO-assigned proteins, and function of these three proteins remain unknown. Functional families for these hypothetical proteins were also analyzed (see Table S3). All of these 33 proteins were allowed for further investigation. The remaining 89 proteins were excluded since these were found to be involved in both pathogen and human metabolic pathways.

**TABLE 2:** Proteins involved in pathogen specific pathways

Serial no.	KO assignment	Accession no.	Protein name	Pathway
1	K18138	tr|Q5ZXL1	Multidrug resistance protein	Beta-lactam resistance; cationic antimicrobial peptide (CAMP) resistance
2	K01928	tr|Q5ZX16	UDP-*N*-acetylmuramoyl-l-alanyl-d-glutamate-2,6-diaminopimelate ligase	Lysine biosynthesis; peptidoglycan biosynthesis
3	K03587	tr|Q5ZX17	Peptidoglycan d,d-transpeptidase FtsI	Peptidoglycan biosynthesis; beta-lactam resistance
4	K05515	tr|Q5ZVR5	Peptidoglycan d,d-transpeptidase MrdA	Peptidoglycan biosynthesis; beta-lactam resistance

### Prediction of subcellular localization.

The shortlisted 33 nonhomologous essential proteins involved in unique pathways of the pathogen were screened through PSORTb, CELLO, and ngLOC servers. A total of 16 proteins were identified as cytoplasmic proteins, and 16 were identified as membrane proteins which were further screened for druggability and antigenicity analysis (see Table S4). However, the study failed to predict the subcellular localization of the hypothetical protein (tr|Q5ZYU2) since these four servers generated different predictions.

### Druggability screening and antigenicity analysis.

Only seven proteins showed similarity with the available drug targets (Table 3) in the DrugBank 5.1.0 database. The remaining 25 showed no hits; these were considered novel drug targets of L. pneumophila, comprising of 13 cytoplasmic and 12 membrane proteins. Antigenicity analysis of membrane protein revealed that only one protein was nonantigenic, whereas the remaining 11 showed antigenicity scores of >0.4 according to the VaxiJen server (Table 4). Furthermore, all the proteins screened after druggability and antigenicity analysis were used for human “anti-target” and microbiome nonhomology studies.

**TABLE 3:** Identified druggable targets with drug names

Serial no.	Accession no.	Protein name	DrugBank ID	Drug name^*a*^	Bit score	Query length (aa)^*b*^
1	tr|Q5ZS52	RNA polymerase sigma factor RpoH	DB08874	Fidaxomicin	103.605	318
2	tr|Q5ZT04	RNA polymerase sigma factor RpoD	DB08226 and DB08266	Myxopyronin B and methyl [(1*E*,5*R*)-5-{3-[(2*E*,4*E*)-2,5-dimethyl-2,4-octadienoyl]-2,4-dioxo-3,4-dihydro-2*H*-pyran-6-yl}hexylidene]carbamate	302.368	248
3	tr|Q5ZUP0	UDP-*N*-acetylmuramate–l-alanyl-gamma-d-glutamyl-meso-2,6-diaminoheptandioate ligase	DB01673, DB03909, and DB04395	Uridine-5′-diphosphate-*N*-acetylmuramoyl-l-alanine,adenosine-5′-[β, γ-methylene]triphosphate, and phosphoaminophosphonic acid-adenylate ester	145.591	398
4	tr|Q5ZXL1	Multidrug resistance protein	DB03825, DB04209, and DB03619	Rhodamine 6G, dequalinium, and deoxycholic acid	427.557	1,020
5	tr|Q5ZX17	Peptidoglycan d,d-transpeptidase FtsI	DB00303 and DB00671	Ertapenem and cefixime	122.094	632
6	tr|Q5ZVR5	Peptidoglycan d,d-transpeptidase MrdA	DB01598, DB01329, DB01327, DB01163, DB01328, DB01413, DB01415, DB00948, DB00438, DB00303, and DB06211	Imipenem, cefoperazone, cefazolin, amdinocillin, cefonicid, cefepime, ceftibuten, mezlocillin, ceftazidime, ertapenem, and doripenem	486.493	612
7	tr|Q5ZZI4	Amino acid permease	DB00123, DB00125, and DB00129	l-Lysine, l-arginine, and ornithine	155.606	424

a Drug names correspond respectively to the DrugBank ID numbers listed in Table 3, column 4.

b aa, amino acids.

**TABLE 4:** Probable antigenic proteins for vaccine targets

Serial no.	Accession no.	VaxiJen v2.0 score	Subcellular localization
1	tr|Q5ZS84	0.4733	Inner membrane
2	tr|Q5ZUM7	0.4869	Inner membrane
3	tr|Q5ZT44	0.5906	Inner membrane
4	tr|Q5ZVR6	0.6058	Inner membrane
5	sp|Q5ZV69	0.6714	Outer membrane
6	sp|Q5ZW98	0.6393	Inner membrane
7	sp|Q5ZSY2	0.4632	Inner membrane
8	sp|Q5ZTN5	0.5461	Inner membrane
9	sp|Q5ZRL2	0.4190	Inner membrane
10	tr|Q5ZY66	0.4935	Outer membrane
11	tr|Q5ZX71	0.5807	Inner membrane

### “Anti-target” and human microbiome nonhomology analysis.

Proteins that are important for human cellular homeostasis and trigger hazardous side effects under the influence of a drug are termed “anti-targets” (53). To avoid severe cross-reaction and toxic effects in humans, identifying nonhomologous proteins as human “anti-targets” is a crucial step. The study revealed that all selected 25 novel therapeutic proteins showed no similarity with 210 human “anti-targets.” In addition, BLASTp analysis of all microbial strains in the Human Microbiome Project (HMP) server carried out using the NCBI blast server revealed that only 5 of 25 proteins showed a similarity of ≤47% (Table 5). Targeting these proteins will be suitable since they are neither involved in common pathways of host-pathogen nor homologous to any human “anti-targets.”

**TABLE 5:** Human microflora nonhomology analysis and subcellular localization

Serial no.	Accession no.	Protein factor	Microbiome similarity	Subcellular localization
1	tr|Q5ZX16	MurE	<46	Cytoplasmic
2	sp|Q5ZUD8	Tig	<45	Cytoplasmic
3	tr|Q5ZS84	MviN	<47	Inner membrane
4	sp|Q5ZW98	Kup1	<47	Outer membrane
5	sp|Q5ZV69	TolB	<44	Inner membrane

### Protein-protein interaction studies.

**FIG 3:**
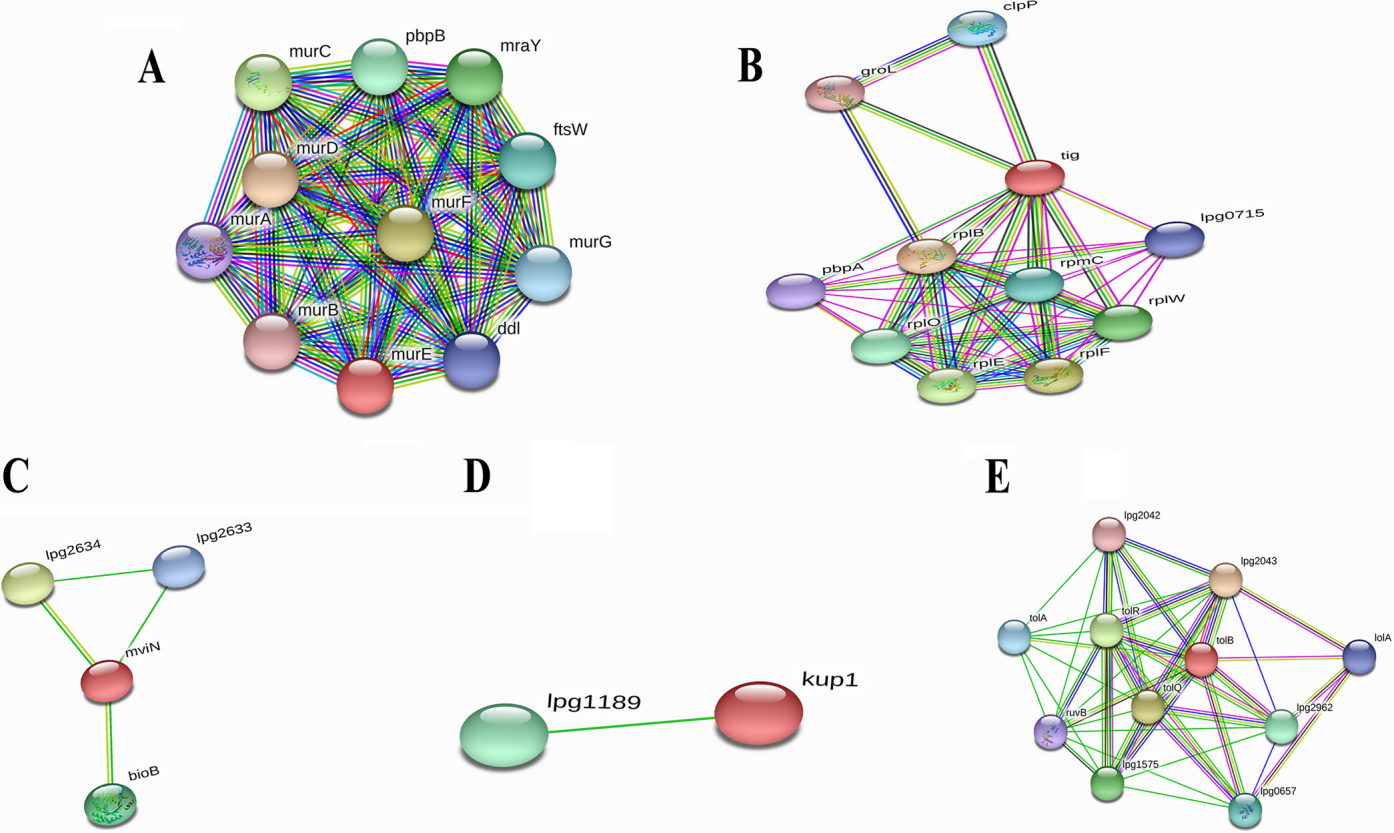
Investigation of PPIs using the STRING v11.5 server. (A) UDP-*N*-acetylmuramoyl-l-alanyl-d-glutamate–2,6-diaminopimelate ligase (*murE*). (B) Trigger factor (*tig*). (C) Lipid II flippase MurJ (*mviN*). (D) Potassium transport system protein Kup1 (*kup1*). (E) Protein TolB (*tolB*).

Among the five novel proteins with ≤47% similarity with human microbiota, three were suggested as novel drug targets for L. pneumophila based on protein-protein interaction (PPI) study (Table 6). PPIs in STRING v11.5 revealed that the cytoplasmic protein UDP-*N*-acetylmuramoyl-l-alanyl-d-glutamate–2,6-diaminopimelate ligase (*murE*) interacted with proteins involved in cell wall biosynthesis pathways (Fig. 3A), whereas “trigger factor (*tig*)” had interactions with proteins that were mainly involved in binding with different rRNA and tRNA molecules (Fig. 3B). Moreover, the inner membrane protein “probable lipid II flippase MurJ (*mviN*)” interacted with proteins that were connected to the peptidoglycan biosynthesis pathway and transportation of lipid-linked peptidoglycan precursors (Fig. 3C), where “biotin synthase (BioB)” is an essential protein for the survival of the pathogen. Important drug targets are listed in Table 6. Another inner membrane protein, “probable potassium transport system protein Kup1 (*kup1*),” presented a single interaction (Fig. 3D), and as a consequence, was excluded from our analysis. Moreover, the remaining outer membrane protein “TolB (*tolB*)” showed close interactions with other proteins (Fig. 3E), mainly found in peptidoglycan synthesis and lipoprotein translocation. Protein TolB also exhibited less similarity (<47%) with human microflora proteins and had an antigenicity score of >0.6, so it was considered a potential vaccine candidate against L. pneumophila. A further study was used for *in silico* vaccine design focusing on protein TolB by a reverse vaccinology approach. Details of PPIs are provided in Table S5 in the supplemental material.

**TABLE 6:** Identified novel drug targets against L. pneumophila

Serial no.	Accession no.	Protein name
1	tr|Q5ZX16	UDP-*N*-acetylmuramoyl-l-alanyl-d-glutamate-2,6-diaminopimelate ligase (*murE*)
2	sp|Q5ZUD8	Trigger factor (*tig*)
3	tr|Q5ZS84	Probable lipid II flippase MurJ (*mviN*)

### Homology modeling, structure optimization, and validation.

The selected single vaccine candidate, protein TolB (Q5ZV69), was modeled to determine three-dimensional (3D) structure through EasyModeller 4.0 (see Fig. S1A). Complete results of template selection for homology modeling are provided in Table S6. The best structure generated for each target was optimized by the ModRefiner server. The refined protein TolB (Q5ZV69) model had a root mean square deviation (RMSD) of 0.1444 and a template modeling (TM) score of 0.9783 in the initial model. After refinement, the Protein Data Bank (PDB) structure of each protein was evaluated using PROCHECK and ERRAT. Psi-Phi pains in Ramachandran plots revealed that protein TolB (Q5ZV69) had 91.9% residues in the most favored regions, 7.8% in the additional allowed regions, and 0.3% in the generously allowed regions (see Fig. S1B). According to ERRAT, the overall quality factor of this protein was 76.1084 (see Fig. S2). An ERRAT score of 50 is usually acceptable, and the value explains the statistics of nonbonded interaction molecules. This indicates that the modeled structure is reliable and stable.

### Antigenic protein selection and T-cell epitope prediction.

From the top membrane proteins, *Legionella pneumophila* (LEGPH) protein TolB (Q5ZV69) was selected based on PPI analysis with a total antigenicity score of 0.6740 for vaccine design against *Legionella*. The ProtParam tool was used to analyze the vaccine proteins’ physicochemical properties (see Table S7). The molecular weight (45.38 kDa) of protein TolB indicated its good antigenic potential. At the same time, the theoretical pI of 8.67 showed that the protein would have a net positive charge below the pI and vice versa. The extinction coefficient was 34,380 at 0.1% absorption, assuming all cysteine residues are reduced. The GRAVY (grand average of hydropathy) value −0.204 described the hydrophilic nature of the protein, whereas the aliphatic index 89.81 ensured the thermostability of the protein. The instability index (38.09) classified the protein as a stable one. In addition, various immunogenic epitopes from LEGPH protein TolB were identified as potential T-cell epitopes that can bind to various HLA-A and HLA-B cells with greater binding affinity. Epitopes that bind to the maximum number of HLA cells were selected.

### Transmembrane topology, antigenicity, population coverage, and allergenicity assessment of T-cell epitopes.

**FIG 4:**
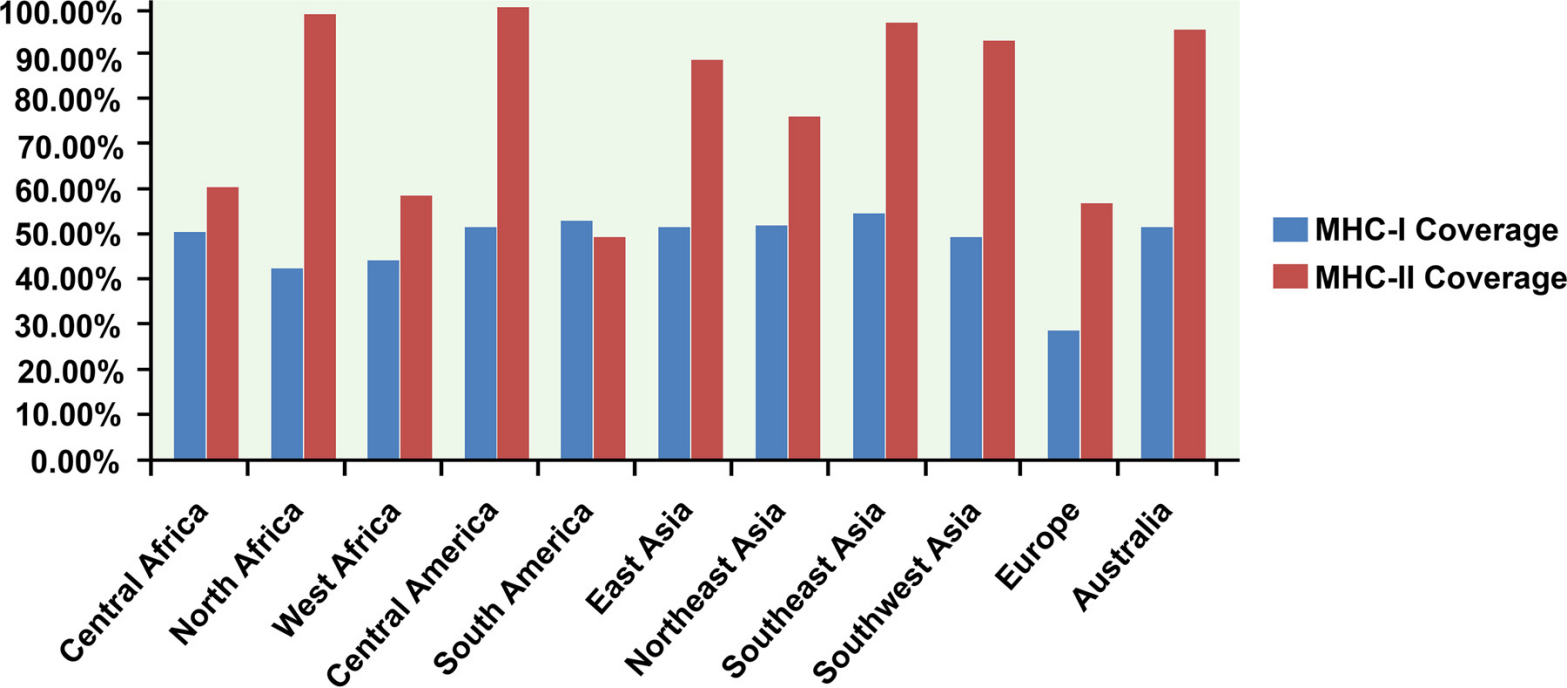
Population coverage analysis of LEGPH protein TolB. Blue indicates MHC-I allele coverage, whereas red indicates MHC-II allele coverage.

The best LEGPH protein TolB epitopes were ranked based on the topology of the transmembrane and the antigenicity score (Table 7). All indicated alleles were identified as optimal binders of the proposed epitopes and used to assess population coverage (Fig. 4). Studies utilizing four separate allergenicity prediction systems have retained epitopes classified as nonallergenic to humans in the predicted epitope list (Table 7).

**TABLE 7:** Predicted T-cell epitopes (MHC-I peptides and MHC-II peptides) of LEGPH protein TolB

Epitope	Length (aa)^*a*^	Antigenicity score	Conservancy (%)	Toxicity	Allergenicity
MHC-I peptides (CTL epitopes)					
IISLFLLLF	9	2.8937	88.89	Nontoxin (–0.82)	Nonallergen
TSGRGGSPQ	9	2.5779	100	Nontoxin (–0.86)	Nonallergen
TSGRGGSPQV	10	2.5666	100	Nontoxin (–0.90)	Nonallergen
SGRGGSPQV	9	2.4721	100	Nontoxin (–0.83)	Nonallergen
IGVQNTGGG	9	2.2694	83.33	Nontoxin (–0.77)	Nonallergen
RIISLFLLLF	10	2.2479	88.89	Nontoxin (–0.91)	Nonallergen
VIALDLELT	9	2.0381	100	Nontoxin (–1.30)	Nonallergen
ISLFLLLFT	9	2.0361	88.89	Nontoxin (–0.99)	Nonallergen
SGRGGSPQVY	10	2.0168	100	Nontoxin (–1.04)	Nonallergen
IISLFLLLFT	10	2.0107	88.89	Nontoxin (–0.82)	Nonallergen
					
MHC-II peptides (HTL epitopes)					
TSGRGGSPQVYRLSL	15	1.8097	100	Nontoxin (–1.42)	Nonallergen
GGRSRYSLEVADADG	15	1.6123	100	Nontoxin (–0.57)	Nonallergen
SGRGGSPQVYRLSLA	15	1.5498	100	Nontoxin (–1.46)	Nonallergen
TFGNSIDTEPRYSPD	15	1.4660	100	Nontoxin (–1.13)	Nonallergen
AREGDVQEPAWSPYL	15	1.4197	88.89	Nontoxin (–0.76)	Allergen
YSLEVADADGHNPQS	15	1.3878	100	Nontoxin (–0.65)	Nonallergen
QLTFGNSIDTEPRYS	15	1.3645	100	Nontoxin (–1.45)	Allergen
PKIYDVDLSSGSMKQ	15	1.3508	72.22	Nontoxin (–0.67)	Nonallergen
LTFGNSIDTEPRYSP	15	1.3006	100	Nontoxin (–1.19)	Nonallergen
YDVDLSSGSMKQLTF	15	1.2975	72.22	Nontoxin (–0.77)	Nonallergen

a aa, amino acids.

### Toxicity and conservancy analysis of T-cell epitope.

ToxinPred server predicted the relative toxicity of the top epitopes (Table 7). Again, putative epitopes generated from LEGPH protein TolB were highly conserved (up to 100%) within specific pathogenic strains. Top epitopes were used to design the final vaccine constructs to allow a broad spectrum of immunity (Table 7).

### B-cell epitope identification and cluster analysis.

B-cell epitopes of the selected protein were generated using a total of six algorithms (Table 8). For envelope glycoprotein, peptide sequences from residues 66 to 77 and residues 404 to 417 were identified as potential B-cell epitopes that could stimulate a preferred immune reaction (see Fig. S3A). Regions from residues 206 to 212 and residues 277 to 287 were more accessible (see Fig. S3B), while amino acid residues 194 to 200 and residues 294 to 300 were considered as possible regions for a beta-turn (see Fig. S3C). Based on Karplus and Schulz’s flexibility prediction, two regions—residues 160 to 166 and residues 308 to 314—were the most flexible (see Fig. S3D). Amino acids in the areas from residues 5 to 23 and from residues 243 to 252 were highly antigenic (see Fig. S3E), while the regions from residues 174 to 180 and from residues 283 to 289 were predominantly hydrophilic (see Fig. S3F). Before the final constructs were designed, peptides containing overlapping epitopes between the top T cells and B cells were identified (see Table S8).

**TABLE 8:** Allergenicity pattern of the predicted B-cell epitopes generated from LEGPH protein TolB

Method	Start	End	Peptide	Length (aa)^*a*^	Allergenicity
BepiPred linear epitope prediction	66	77	SGPQGANSQSSV	12	Nonallergen
	404	417	AREGDVQEPAWSPY	14	Allergen
Chou and Fasman beta turn prediction	194	200	SWSPNGK	7	Nonallergen
	294	300	SGRGGSP	7	Nonallergen
Emini surface accessibility prediction	206	212	SFEKKKA	7	Nonallergen
	277	287	IDTEPRYSPDG	11	Nonallergen
Karplus and Schulz flexibility prediction	160	166	QRNGGRS	7	Allergen
	308	314	ADGQISR	7	Nonallergen
Kolaskar and Tongaonkar antigenicity scale	5	23	IISLFLLLFTGQVIALDLE	19	Nonallergen
	243	252	GQHLAVVLSK	10	Nonallergen
Parker hydrophilicity prediction	174	180	DADGHNP	7	Allergen
	283	289	YSPDGRS	7	Nonallergen

a aa, amino acids.

### Design of the vaccine construct.

Both clusters and singletons (which contain a single epitope) were used to construct the final vaccine molecules. Three adjuvants—beta-defensin, ribosomal protein L7/L12, and Mycobacterium tuberculosis HABA protein (AGV15514.1)—were used to construct vaccines. The adjuvants were added at the N-terminal end of the vaccine construct, which helps the vaccine to create a more robust immune response in the patient’s body. The EAAAK, GGGS, GPGPG, and KK linkers were used to connect the epitopes in the correct position. The PADRE sequence was conjugated with the vaccine construct to ensure the maximum major histocompatibility complex class II (MHC-II) allele coverage. Three constructs with 476 (V1), 561 (V2), and 590 (V3) amino acid residues were designed and used for analysis (Table 9). A schematic representation of the constructed vaccine is shown in Fig. S4 in the supplemental material.

**TABLE 9:** Allergenicity prediction and antigenicity analysis of designed vaccine constructs

Vaccine construct	Composition	Complete sequence of vaccine construct	Allergenicity	VaxiJen score (threshold 0.4)
V1	Predicted CTL, HTL, and BCL epitopes of LEGPH protein TolB with β defensin adjuvant and PADRE sequence	EAAAKGIINTLQKYYCRVRGGRCAVLSCLPKEEQIGKCSTRGRKCCRRKKEAAAKAKFVAAWTLKAAAGGGSRIISLFLLLFTGGGSTSGRGGSPQVYGGGSQVIALDLELTQGGGSIGVQNTGGGPGGGSLPAREGDVQGGGSDVDLSSGSMKGGGSTFGNSIDTEGPGPGTPKIYDVDLSSGSMKQLTFGGPGPGLTFGNSIDTEPRYSPDGGPGPGFTSGRGGSPQVYRLSLAGPGPGYSLEVADADGHNPQSGPGPGRVTFEGNYNARASYTGPGPGGGRSRYSLEVADADGGPGPGISGPQGANSQSSVSTGPGPGVIALDLELTQGINSAKKSGPQGANSQSSVKKAREGDVQEPAWSPYKKSWSPNGKKKSGRGGSPKKSFEKKKAKKIDTEPRYSPDGKKADGQISRKKIISLFLLLFTGQVIALDLEKKGQHLAVVLSKKKYSPDGRSKKAKFVAAWTLKAAAGGGS	Nonallergen	0.6034
V2	Predicted CTL, HTL, and BCL epitopes of LEGPH protein TolB with L7/L12 ribosomal protein adjuvant and PADRE sequence	EAAAKMAKLSTDELLDAFKEMTLLELSDFVKKFEETFEVTAAAPVAVAAAGAAPAGAAVEAAEEQSEFDVILEAAGDKKIGVIKVVREIVSGLGLKEAKDLVDGAPKPLLEKVAKEAADEAKAKLEAAGATVTVKEAAAKAKFVAAWTLKAAAGGGSRIISLFLLLFTGGGSTSGRGGSPQVYGGGSQVIALDLELTQGGGSIGVQNTGGGPGGGSLPAREGDVQGGGSDVDLSSGSMKGGGSTFGNSIDTEGPGPGTPKIYDVDLSSGSMKQLTFGGPGPGLTFGNSIDTEPRYSPDGGPGPGFTSGRGGSPQVYRLSLAGPGPGYSLEVADADGHNPQSGPGPGRVTFEGNYNARASYTGPGPGGGRSRYSLEVADADGGPGPGISGPQGANSQSSVSTGPGPGVIALDLELTQGINSAKKSGPQGANSQSSVKKAREGDVQEPAWSPYKKSWSPNGKKKSGRGGSPKKSFEKKKAKKIDTEPRYSPDGKKADGQISRKKIISLFLLLFTGQVIALDLEKKGQHLAVVLSKKKYSPDGRSKKAKFVAAWTLKAAAGGGS	Nonallergen	0.5524
V3	Predicted CTL, HTL, and BCL epitopes of LEGPH protein TolB with HABA adjuvant and PADRE sequence	EAAAKMAENPNIDDLPAPLLAALGAADLALATVNDLIANLRERAEETRAETRTRVEERRARLTKFQEDLPEQFIELRDKFTTEELRKAAEGYLEAATNRYNELVERGEAALQRLRSQTAFEDASARAEGYVDQAVELTQEALGTVASQTRAVGERAAKLVGIELEAAAKAKFVAAWTLKAAAGGGSRIISLFLLLFTGGGSTSGRGGSPQVYGGGSQVIALDLELTQGGGSIGVQNTGGGPGGGSLPAREGDVQGGGSDVDLSSGSMKGGGSTFGNSIDTEGPGPGTPKIYDVDLSSGSMKQLTFGGPGPGLTFGNSIDTEPRYSPDGGPGPGFTSGRGGSPQVYRLSLAGPGPGYSLEVADADGHNPQSGPGPGRVTFEGNYNARASYTGPGPGGGRSRYSLEVADADGGPGPGISGPQGANSQSSVSTGPGPGVIALDLELTQGINSAKKSGPQGANSQSSVKKAREGDVQEPAWSPYKKSWSPNGKKKSGRGGSPKKSFEKKKAKKIDTEPRYSPDGKKADGQISRKKIISLFLLLFTGQVIALDLEKKGQHLAVVLSKKKYSPDGRSKKAKFVAAWTLKAAAGGGS	Nonallergen	0.5726

### Allergenicity, antigenicity, physicochemical properties, and secondary structure analysis of vaccine constructs.

The results revealed construct V1’s superiority due to a greater antigenicity score (0.603) and nonallergenic behavior (Table 9). The final construct of the vaccine was characterized by its physical and chemical properties. Construct V1’s molecular weight was 48.34 kDa, while the theoretical pI was measured at 9.78, indicating that the protein should have a net negative charge over the pI and vice versa. The half-life of the vaccine was expected to be more than 10 h in Escherichia coli *in vivo*. The estimated extinction rate and an aliphatic index were 41,370 and 61.74, respectively. The protein’s computed GRAVY value was −0.545, while the instability index (33.51) classified the protein as stable. Constructed V1 was, in contrast, characterized by a 6.30% alpha-helix, a 24.78% sheet, and a 68.92% coil structure (see Fig. S5).

### Vaccine tertiary structure prediction, refinement, validation, and disulfide engineering.

**FIG 5:**
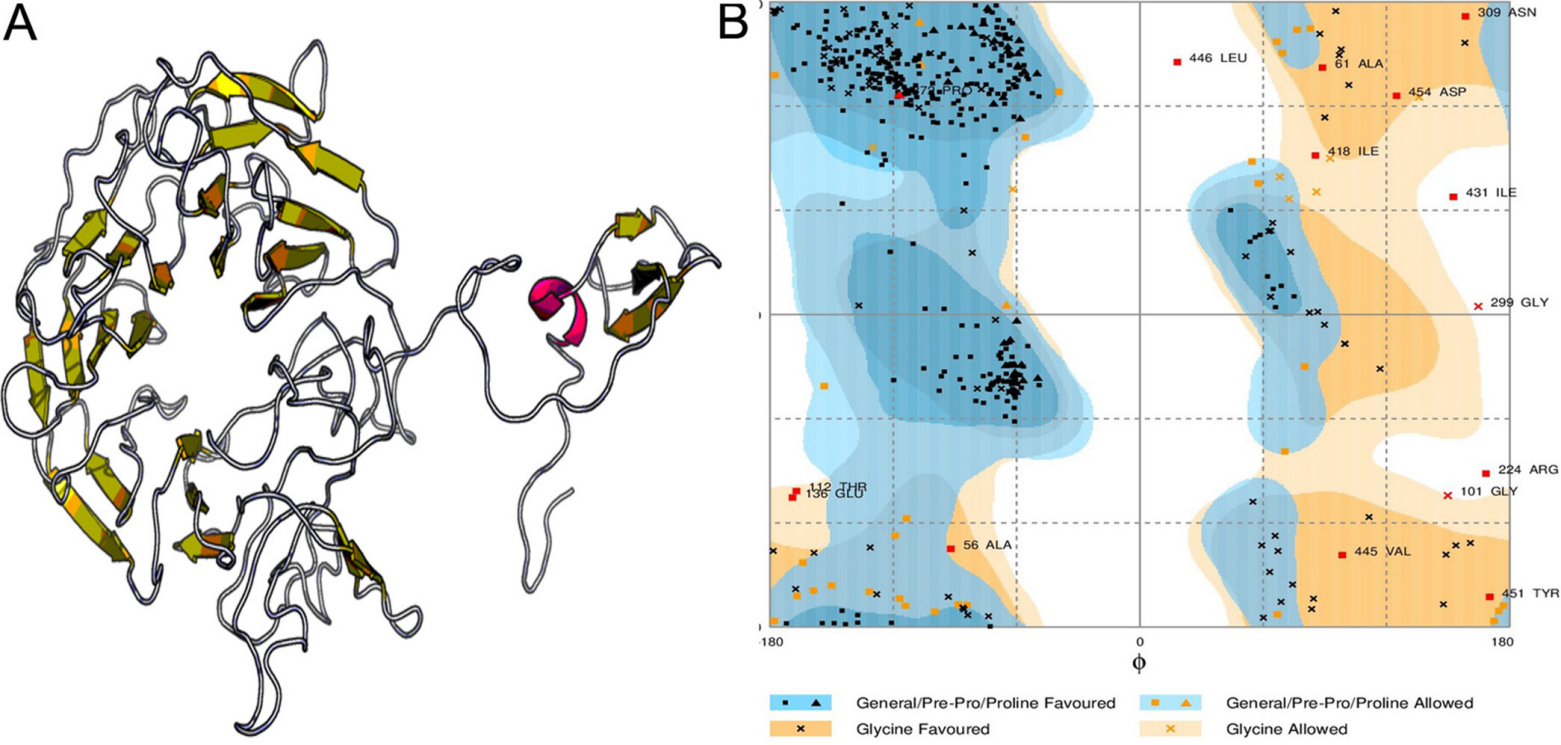
Tertiary structure prediction and validation of vaccine protein V1. (A) Structure diagram. (B) Validation of the 3D structure of vaccine protein V1 as determined by Ramachandran plot analysis.

**FIG 6:**
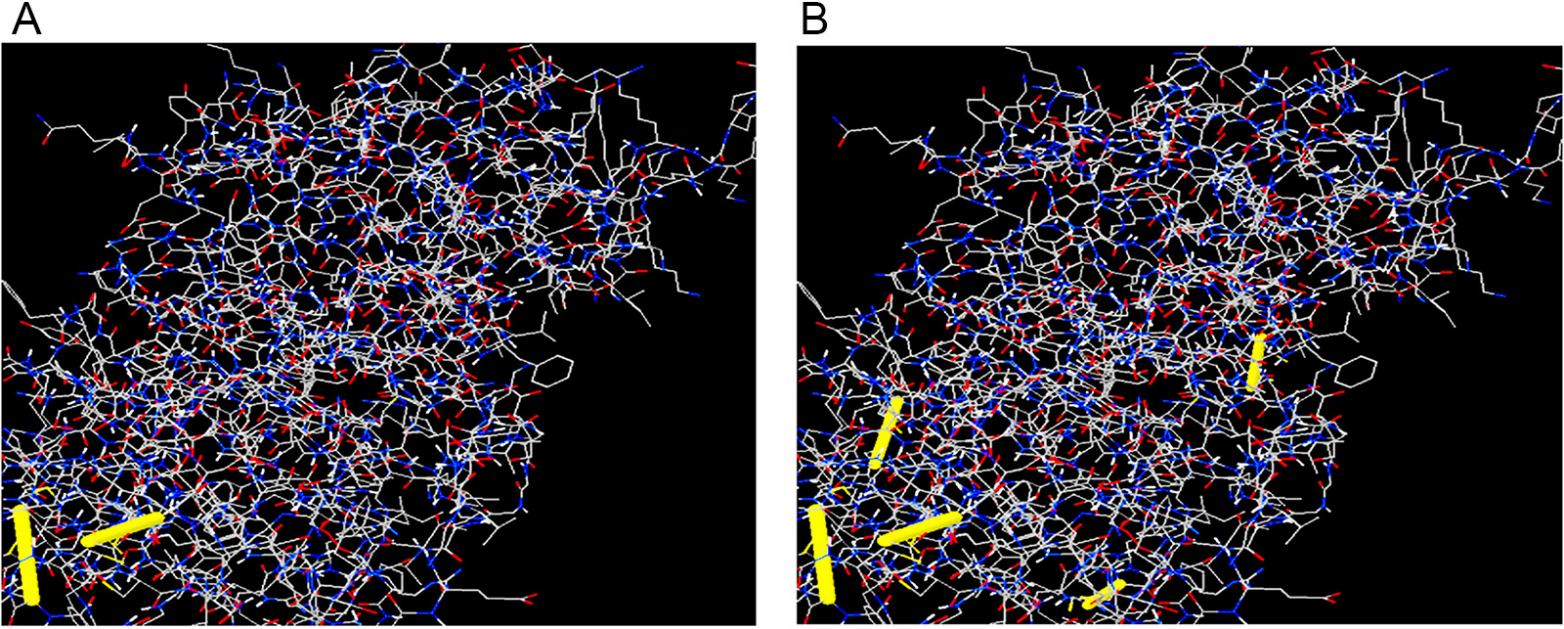
Disulfide engineering of vaccine protein V1. (A) Initial form (two disulfide bonds, indicated by yellow bands). (B) Mutant form (five disulfide bonds, indicated by yellow bands).

2YMU (chain A) from the PDB database was detected as the most suitable V1 template, and a single-domain 3D model was created through the Raptor X server (Fig. 5A). All 476 residues were modeled by the server, while only 8% of residues were in the disordered region. The 3D model’s *P* value was 5.30^e−11^, which ensured better-quality modeling of the proposed vaccine. Of the residues, 81.4, 11.8, and 6.8% were in the favored, allowed, and outlier regions, respectively, prior to refinement. However, 88.4% of the residues were in the favored region after refining. A Ramachandran plot showed that 8.4%- and 3.2% of the residues were in the allowed and outlier regions (Fig. 5B). Homology modeling of constructs V2 and V3 was also performed, as shown in Fig. S6. For construct V1, 71 amino acid pairs could shape disulfide bonds. However, three pairs—ASN 202/GLY 226, SER 211/GLY 217, and ASN 309/LEU 317—based on the chi3 and B-factor values met disulfide bond-forming requirements (Fig. 6).

### Protein-protein docking, molecular dynamics simulation, and interaction analyses of vaccine constructs and TLR-2.

**FIG 7:**
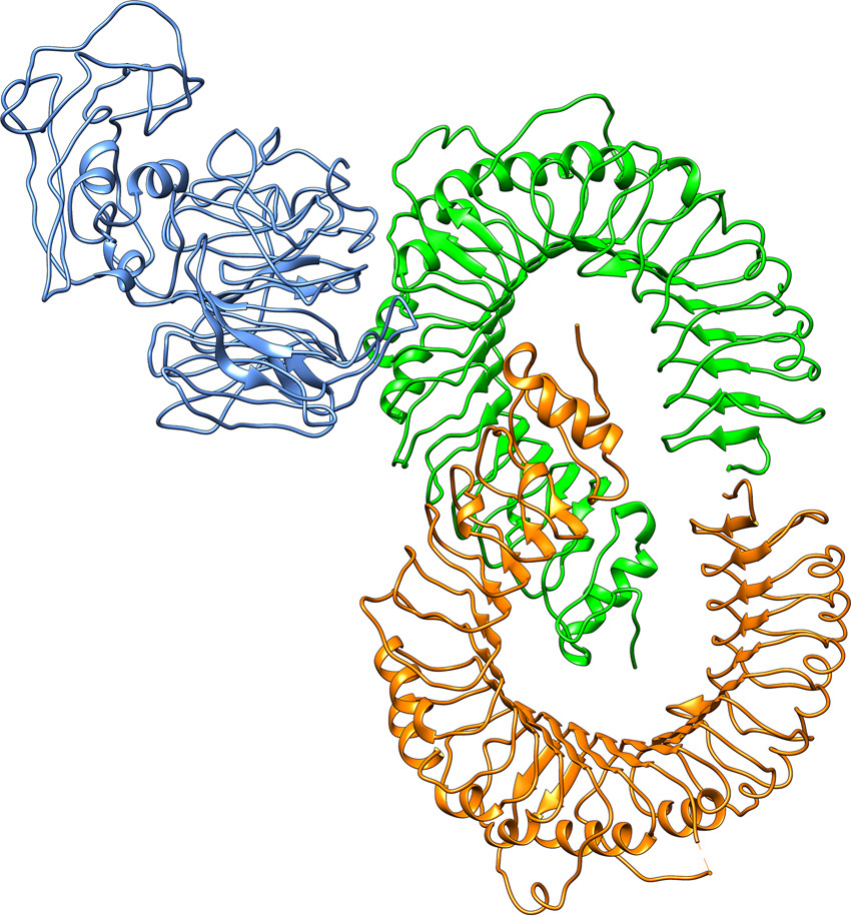
Docked complex of vaccine construct V1 with human TLR-2. Chain A of TLR-2 is indicated in orange, chain B of TLR-2 is indicated in green, and the vaccine molecule is indicated in blue.

Docking’s study revealed that construct V1 bound in the groove of different HLAs with minimal binding energy, which was biologically significant (Table 10). The predicted binding energies for the vaccine V1-TLR-2 complex were −1,232.8 and −275.55 kJ/mol as determined by ClusPro (Fig. 7) and the HDOCK server, respectively. FireDock output refinement of PatchDock server showed the lowest global energy of −15.64 for solution 2. The lowest binding energy measured the highest binding affinity between TLR-2 and vaccine construct. The molecular dynamics simulation of the vaccine onto the TLR-2 protein was performed for 100 ns.

**TABLE 10:** Docking scores of vaccine construct V1 with different HLA alleles^*a*^

HLA allele PDB ID	Global energy	Hydrogen bond energy	ACE	Score	Area
1A6A	–12.98	–4.93	7.18	17648	3,028.30
1H15	–30.47	–5.55	5.82	16608	2,414.80
2Q6W	–10.99	–2.58	7.65	17294	2,662.20
2SEB	–27.99	–8.91	0.83	18036	2,334.50
2FSE	–1.43	–0.46	0.05	19568	2,748.70
3C5J	–9.43	–3.63	15.90	16902	2,337.90

a These alleles included HLA-DRB1*03:01 (1A6A), (HLA-DRB5*01:01 (1H15), HLA-DRB3*01:01 (2Q6W), HLA-DRB1*04:01 (2SEB), HLA-DRB1*01:01 (2FSE), and HLA-DRB3*02:02 (3C5J). ACE, atomic contact energy.

**FIG 8:**
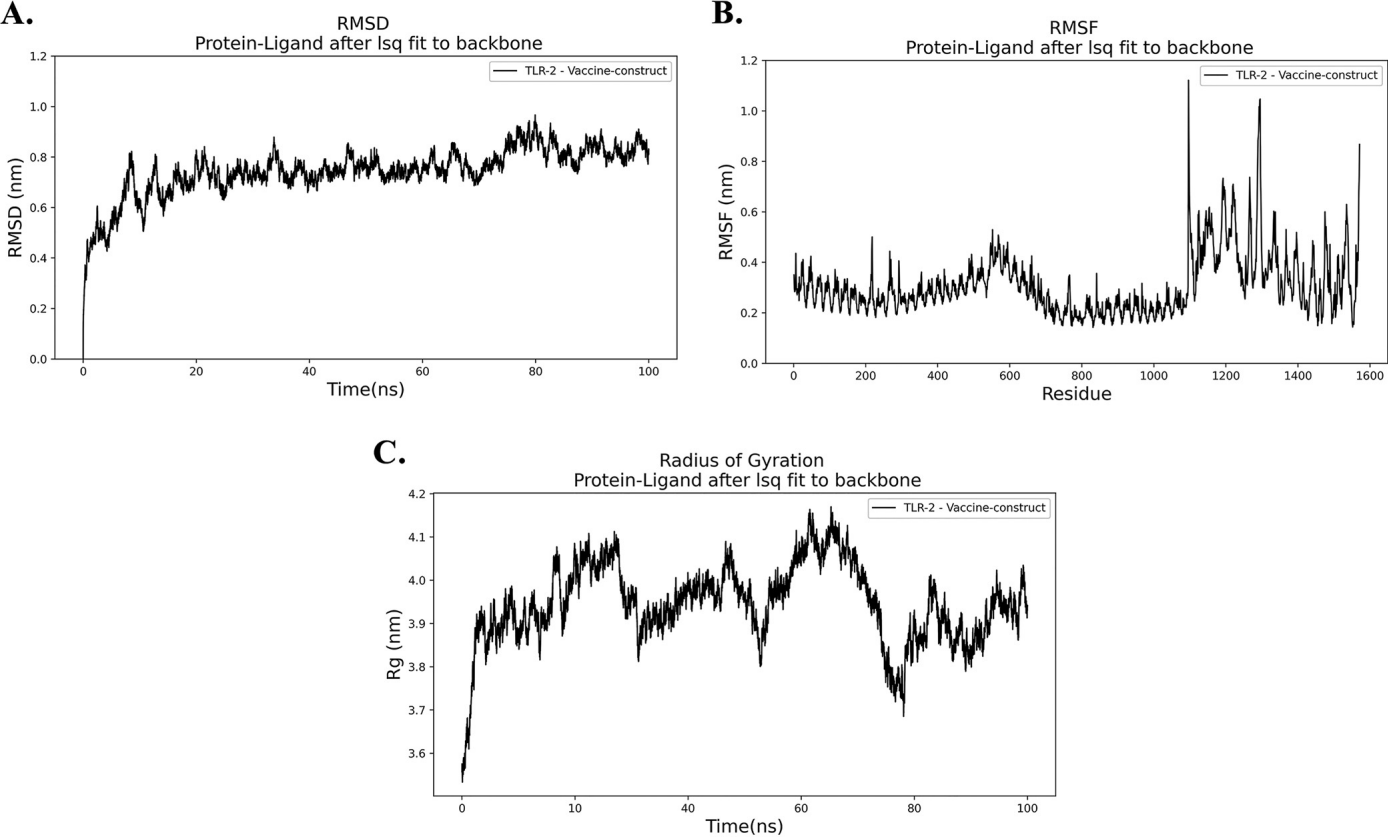
Molecular dynamics simulation of the vaccine protein V1/TLR-2 complex. (A) RMSD, root mean square deviation. (B) RMSF, root mean square fluctuation. (C) Rg, radius of gyration.

**FIG 9:**
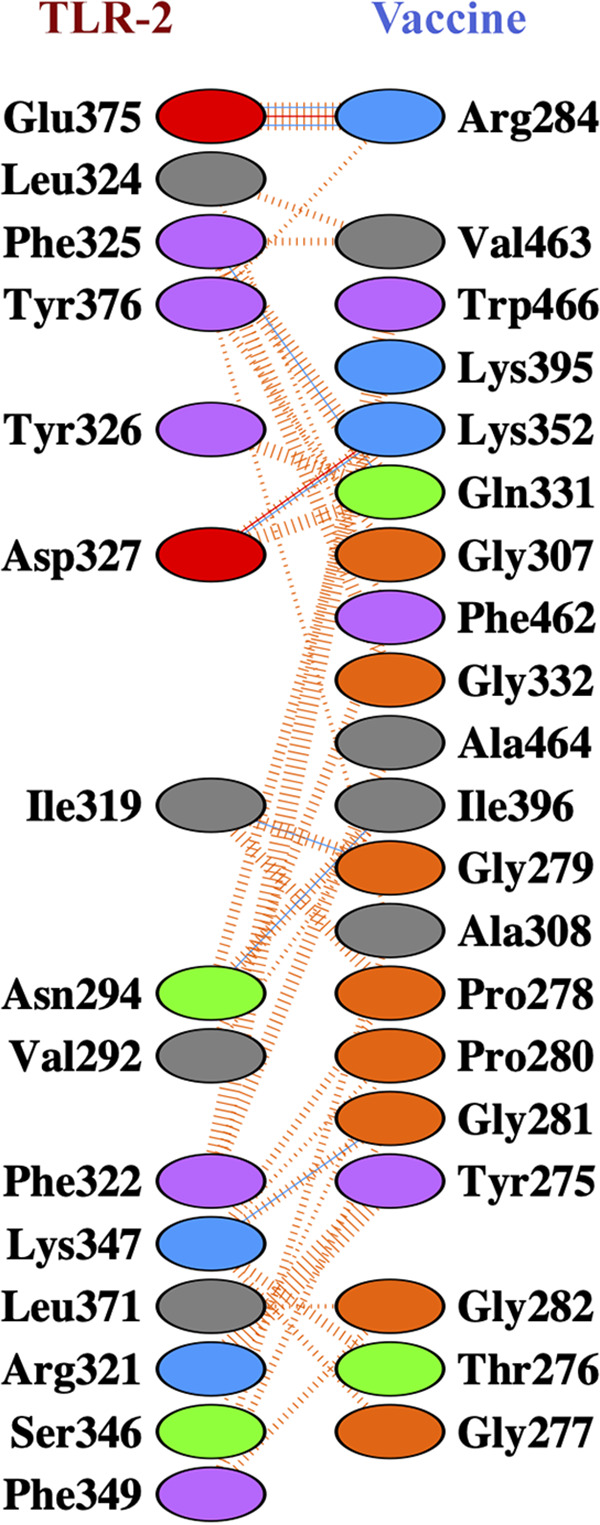
Vaccine construct V1-TLR-2 docked complex. Interacting residues are obtained after a 100-ns molecular dynamics simulation. Blue lines indicate hydrogen bonds, red lines indicate salt bridges and dashed orange lines indicate nonbonding interaction between the V1-TLR-2 complex.

The trajectory of the molecular dynamics (MD) simulation of the docked complex was analyzed to plot RMSD, root mean square fluctuation (RMSF), and radius of gyration (Rg) plots. RMSD plot (Fig. 8A) of the simulation trajectory calculated by referencing the backbone of the complex. The plot showed a minor deviation from the initial structure, indicating the docked complex’s stability. Aside from the initial increase, in the beginning, the RMSD plot never crossed the 0.8 nm of deviation and stayed between 0.5 and 0.8 nm. The RMSF plot usually shows the fluctuations in the residues of a protein complex. The RMSF plot of the complex showed a change in the flexible regions of the receptor and minor fluctuation in the vaccine part (Fig. 8B). The Rg plot analyzes the compactness of a complex. The docked complex of the vaccine onto the receptor shows significant compactness suggesting adhesion onto the receptor (Fig. 8C). Docked complex (vaccine and chain B of TLR-2) produced seven hydrogen bond interactions and two salt bridges (Fig. 9; see also Table S9). These simulation and interaction analyses showed significant evidence of the stability of the receptor vaccine complex, suggesting that a proper immune response might occur for docking the vaccine onto the receptor complex.

### Codon adaptation for *in silico* cloning and expression in *E. coli*.

The optimized codon adaptation index was 0.95, and the GC content of that sequence was 54.01%. These results showed better expression in E. coli K-12. The optimized codons were introduced into vector pET28a(+) and the restriction sites HindIII and BamHI. A 6,346-bp clone was created [see Fig. S7; the desired 1,434-bp sequence of the pET28a(+) vector sequence is indicated in red].

## DISCUSSION

Among all the pathogenic agents responsible for pneumonia and respiratory tract infection, L. pneumophila is one of the main causative agents showing high resistance against many antibiotics. Although clinical studies of antibiotic treatment against Legionnaires’ disease have been reported, the number of such cases is insufficient (54). Hence, treatment recommendations are limited due to a lack of evidence (55). However, Rahimi and Vesal reported that L. pneumophila strains demonstrated the greatest resistance to ciprofloxacin, erythromycin, clarithromycin, moxifloxacin, and azithromycin (56). Therefore, the primary goal here was to screen out and determine novel drug targets and vaccine candidates for L. pneumophila through subtractive genomics and a reverse vaccinology approach.

Computational predictions help identify essential proteins for the pathogen’s survival, nonhomologous to host proteins, and no involvement in the metabolic pathways of the host, leading to choosing the proteins only present and involved in pathogen-specific metabolic pathways. The complete reference proteome of L. pneumophila (2,930 proteins) was searched and downloaded from the NCBI protein database. Proteins involved in several common cellular systems emerged as homologous with the same functions between humans and bacteria during evolution (57, 58); these were removed in this study based on their identity with human proteins. Essential proteins are most promising for new antibacterial drug targets since most antibiotics are designed to bind essential gene products (59) and can be considered pathogen-specific drug targets (60). The study revealed 125 unique, essential proteins of L. pneumophila that can be considered suitable drug targets. Localization is an important aspect of any possible drug target since the cellular functions of proteins are often localized in specific compartments of the cell; hence, studying subcellular localization will be helpful in understanding disease mechanisms, as well as in developing new vaccine candidates and drug targets. Although both membrane and cytoplasmic proteins could serve therapeutic targets (61), membrane proteins have been mostly reported for vaccine candidates (62). Hence, we used membrane proteins for vaccine construction, whereas cytoplasmic proteins were proposed as suitable drug targets. Again, the usage of antibiotics has already reduced their efficiency due to gene mutation and, thereby, the rapid emergence of resistant bacteria (63). Antibiotic and antimicrobial resistance crises have been observed due to the misuse and overuse of these drugs and to the scarcity of developing new drugs by the pharmaceutical industry (61, 64). Broad-spectrum drugs medicated for a pathogen or group of the pathogen may cause mutational changes and enhance the transfer of genes to other pathogens, which can show resistance to drugs, leading to the emergence of resistant bacteria. To avoid these problems, the selection of novel drug targets and vaccine candidates is a must; therefore, shortlisted 32 proteins were further screened through the DrugBank 5.1.0 database with default parameters.

Antigenicity scores for selected 12 novel drug-targeted membrane proteins revealed that only one protein was nonantigenic, whereas the remaining 11 showed antigenicity scores of >0.4. These 11 proteins can be used to design B-cell and T-cell epitopes (specific for CTL and HTL) in the future to lessen the disease caused by L. pneumophila. Several drugs from different categories, such as nonnarcotic analgesics, nonsteroidal anti-inflammatory drugs, antidepressants, and vasodilators, were withdrawn from 1960 to 1999 due to various health-related cross-reactivity causing hepatic, cardiovascular, dermatological, and hematologic toxicities, as well as due to carcinogenic effects (65). For example, bromfenac (66), ebrotidine (67), and trovafloxacin (68) were withdrawn from pharmaceutical markets worldwide since they showed hepatotoxic effects. Therefore, specific recognition must be maintained by an ideal drug target to the drug treated against it; furthermore, this drug target needs to be significantly different from the host proteins. To avoid severe cross-reactions and adverse effects in humans, identifying nonhomologous proteins as human “anti-targets” (also referred to as essential proteins) was a crucial step in this study. Targeting human microbiome nonhomology proteins will be suitable since they are neither involved in common host-pathogen pathways nor homologous to any human “anti-targets.” Furthermore, drugs or vaccines designed and administered for these novel targets will be less harmful to other commensal microbial strains dwelling in different body sites of a healthy human. The study revealed that most cytoplasmic proteins are involved in cell wall biosynthesis pathways and are essential for cell division. However, three proteins—UDP-*N*-acetylmuramoyl-l-alanyl-d-glutamate-2,6-diaminopimelate ligase, trigger factor, and probable lipid II flippase MurJ—influence other crucial interacting proteins of the pathogen (Fig. 3) and were thus identified as the most attractive drug targets that can lead to the development of suitable therapeutics in the future. This study’s predicted single vaccine candidate, TolB (Q5ZV69), was analyzed to develop a potential and highly immunogenic vaccine candidate against *L. pneumophila*. Tol proteins are crucial for the survival and pathogenicity of many bacterial pathogens, such as Escherichia coli, Haemophilus ducreyi, Vibrio cholerae, and Salmonella enterica (69, 70). Protein TolB is involved in the translocation of the toxin across the outer membrane (69, 71). TolB is crucial in the Tol-Pal system since it directly interacts with Pal and other outer membrane proteins required for bacterial pathogenesis (72, 73). Apart from colicin uptake, the Tol-Pal system has a role in cell growth and fitness. *tolB* is part of the Tol-Pal system found in most Gram-negative bacteria that contains *tolA*, *tolQ*, *tolR*, and *pal* (74). Tol-Pal mutants contain potential vaccine properties and have promising therapeutic potential against multidrug-resistant bacterial pathogens (75). It was observed that the *tolB* mutant induces IgG production and gives a higher immunity level than do *tolA* and *tolR* mutants (76). Therefore, therapeutic inactivation of the TolB protein may affect and disrupt the interactions and synchronization of TolA, TolQ, TolR, and Pal proteins. The utilization of whole protein as a vaccine candidate is associated with the risk of the emergence of virulence property. Subunit vaccines, in contrast, can overcome such problems and consist of only the antigenic part of the pathogen with the potential to induce protective immunity within the host. Various antigenic epitopes were generated using different bioinformatics tools, which were extensively investigated for immunogenicity, toxicity profile, allergenicity pattern, and conservancy analysis. The PADRE sequence reportedly decreased the polymorphisms of HLAs in different populations (77, 78). In previous *in vivo* research, the linkers improved the immunogenicity of the vaccines (79, 80). Therefore, here, these critical considerations were considered in developing the final vaccine model. Moreover, the results of docking analysis revealed the binding affinity of promiscuous epitopes with different HLA alleles. In addition, the interaction between construct V1 and Toll-like receptor 2 (TLR-2) was checked to demonstrate the used adjuvants’ efficacy. TLR-2 is a major mammalian TLR recognizing lipoproteins derived from bacteria, viruses, fungi, and parasites (81). Literature reports suggest that TLR-2-mediated recognition of *Legionella* LPS and subsequent chemokine-dependent cellular recruitment is a crucial host innate response in L. pneumophila pneumonia (82). Therefore, we chose TLR-2 to evaluate its binding interaction with protein TolB, and our result revealed higher binding affinity, which is biologically significant. The vaccine-receptor complex also showed minimum deformability as well at the molecular level. Human *Legionella* infection has been studied in different animal models, most notably in mice and guinea pigs (83, 84). However, it was observed that the majority of mouse strains are resistant to *Legionella* infection and disease progression (85). Although the guinea pig model response to *Legionella* is closely similar to human infection, the scarcity of mutants and the high cost of specific immunological reagents discourage its use (86). Other animal models, such as Caenorhabditis elegans (87), Drosophila melanogaster (88), wax moth (89), and amoebae (90), have been developed to study *Legionella* infection. However, these models are not popular for different limitations (87, 88). Hence, the weakness of the existing models is a challenge to validate the vaccine target.

In conclusion, by using a subtractive genomics and reverse vaccinology approach, we are trying to develop novel therapeutics against L. pneumophila and may help to reduce the rate of both mortality and morbidity caused by this pathogen. We mainly considered the key that is a must for survival of the pathogen, nonhomologous to the human, as well as human microbiota, and screened out B- and T-cell epitopes of OMPs. The highest scoring OMPs and epitopes will facilitate future wet lab-based experiments to develop drugs and suitable vaccine candidates against intracellular L. pneumophila infection. After intensive analysis, only three proteins were identified and proposed as novel therapeutic targets against L. pneumophila. Only OMP TolB was identified as a potential vaccine candidate with a better antigenicity score. However, we suggest further *in vitro* and *in vivo* laboratory trials to validate our prediction.

## MATERIALS AND METHODS

### Proteome retrieval and finding paralogous sequences.

The entire proteome of L. pneumophila subsp. *pneumophila* (strain Philadelphia 1/ATCC 33152/DSM 7513) (Proteome ID UP000000609), was retrieved from UniProt containing total 2930 proteins (91) which were subjected to CD-Hit analysis (http://weizhong-lab.ucsd.edu/cdhit-web-server/cgi-bin/index.cgi) (92). The sequence identity cutoff score was set at 0.6 to exclude redundant sequences of >60% identity through this method. Only nonparalogous protein sequences, excluding the redundant sequences (paralogous), were passed through further study.

### Identification of human nonhomologous proteins.

The goal of the nonhomology analysis of L. pneumophila subsp. *pneumophila* is to identify pathogen-specific proteins that are nonhomologous to the human. BLASTp analysis was carried out on nonparalogous proteins against the reference proteome of Homo sapiens using a threshold expectation value (E value) of 10^−3^ through Ensembl (https://uswest.ensembl.org/Multi/Tools/Blast?db=core) (93). Proteins that showed significant hits were filtered out, meaning they had similarities with the human genome leaving just the nonhomologous sequences. These nonhomologous sequences were screened. This step aims to avoid any functional resemblance with human proteomes and reduce any unwanted cross-reactivity of the drugs, thereby preventing the binding of drugs to the active site of homologous human proteins (94).

### Identification of essential nonhomologous protein.

Nonhomologous proteins were subjected to the DEG (59). DEG contains 53,885 essential genes and 786 essential noncoding sequences. BLASTp of previously selected nonhomologous proteins was carried out by selecting all the organisms present in DEG 15.2 server and using a threshold expectation value 10^−10^ and a minimum bit-score cutoff of 100 as parameters, but the hits with a E value of ≤10^−100^, an identity of ≥25%, and the same annotated function of the query were selected as essential proteins. These essential proteins were further subjected to metabolic pathway analysis.

### Metabolic pathway analysis and family prediction of hypothetical proteins.

The Kyoto Encyclopedia of Genes and Genomes (KEGG) includes complete metabolic pathways in living organisms (95). Using the three-letter KEGG organism codes “has” and “lpn” for human and L. pneumophila subsp. *pneumophila*, respectively, all of the metabolic pathways present in the host (H. sapiens) and the pathogen were collected separately. A manual comparison was made to identify the metabolic pathways only present in the pathogen as unique to L. pneumophila subsp. *pneumophila* using the KEGG pathway database (96), whereas the remaining pathways were grouped as common. The predicted human nonhomologous, essential proteins of L. pneumophila subsp. *pneumophila* were screened by BLASTp through KAAS (KEGG Automatic Annotation Server) (97) at KEGG to identify potential drug and vaccine targets. KAAS generates functional annotation of genes by BLAST comparisons against the manually compiled KEGG GENES database, and metabolic proteins are listed by assignments of KO (KEGG Orthology). It automatically generates KEGG pathways that indicate specific metabolic proteins. For the next phase of the subcellular localization study, proteins involved in these particular metabolic pathways of the pathogen and proteins allocated to KO but not involved in specific pathways were identified, except for proteins involved in common human and pathogen pathways. SVMProt is a server that uses a support vector machine to identify a protein sequence from its primary sequence into a specific class that contains all major groups of enzymes, transporters, receptors, pathways, and RNA- and DNA-binding proteins. The SVMProt server (98) was used to determine the functional classes of the pathogen’s specific metabolic proteins in all uncharacterized, hypothesized proteins.

### Prediction of subcellular localization.

Because of the Gram-negative cell wall structure, L. pneumophila subsp. *pneumophila* proteins can be identified in five viable subcellular locations: cytoplasm, inner membrane, periplasm, outer membrane, and extracellular. Cytoplasmic proteins can be used as targets for medications, while surface membrane proteins can be used for both the medication and vaccine (99). PSORTb v3.0.2 (http://www.psort.org/psortb/index.html) (100), CELLO v.2.5 (http://cello.life.nctu.edu.tw/) (101), and ngLOC (102) were used to predict the locations of selected nonhomologous essential pathogen proteins. The best score for a location was counted and generated by these servers. The final locations of these proteins were assessed via three steps: (i) if all three servers predicted the same location for a protein, that location was selected as the final result; (ii) if any two of these three servers predicted the same location for a protein, that location was selected as the final result; and (iii) if they predicted three different locations for a protein then PSLpred server (https://webs.iiitd.edu.in/raghava/pslpred/index.html) (103) was used and the predicted location for that protein was matched with previous results predicted by PSORTb v3.0.2, CELLO v.2.5, and ngLOC servers.

### Druggability screening and antigenicity analysis.

DrugBank Database 5.1.0 (104) contains 2,556 approved small molecule drugs, 962 licensed pharmaceutical drugs, 112 nutraceuticals, and more than 5,125 experimental drugs. In addition, 5,117 nonredundant protein sequences (i.e., drug target/enzyme/carrier/transporter) are linked to these drug entries. A “druggable” target must potentially bind to drugs and drug-like molecules with high affinity. Shortlisted cytoplasmic and membrane proteins were screened through the database of DrugBank 5.1.0 using default parameters. The drug targets from the shortlisted cytoplasmic and membrane proteins in the DrugBank database indicate that the same biological functions can act as evidence for their druggable properties and be grouped as current therapeutic targets. In contrast, their absence indicated those drug targets’ novelty and thus are classified as “novel therapeutic targets”. A total of 45 proteins that showed no similarity after passing through the DrugBank database were listed as novel drug targets and vaccine candidates. VaxiJen v2.0 (105) was used to predict protective antigens and subunit vaccines. Only membrane protein sequences of novel L. pneumophila subsp. *pneumophila* drug targets were subjected to VaxiJen v2.0, selecting a threshold value of 0.4. Proteins that showed antigenicity prediction >0.4 in VaxiJen v2.0 were transferred for the next step as potential vaccine candidates.

### “Anti-target” analysis of the essential, nonhomologous, and novel drug targets.

For humans, the human ether-a-go-go-related gene (hERG), constitutive androstane receptor (CAR), pregnane X receptor (PXR), and P-glycoproteins (P-gp) are anti-targets or alternative drug targets for host protein candidates. Some receptive membranes are also classified as “anti-target.” They are adrenergic α1a, dopaminergic D2, muscarinic M1, and serotonergic 5-HT2C. A total of 210 human “anti-targets” have been identified in the literature (106), and the corresponding sequences of these proteins have been obtained from the NCBI protein database. BLASTp analysis was carried out for all nonhomologous, essential “novel drug target” proteins against these “anti-targets,” setting an E value threshold of <0.005, a query length of >30%, and an identity of <30% as parameters. Nonhomologous proteins showing <30% identity against these “anti-target” proteins were transferred to the next step.

### Human microbiome nonhomology analysis.

The correlation between gut flora and humans is not merely commensal but a symbiotic, mutualistic relationship (107), and different beneficial functions of the human microbiome were also reported (108, 109). Unintentional blocking or accidental inhibition of proteins present in this microflora may lead to adverse effects in the host (110). Screening of nonhomologous, essential proteins selected as vaccine candidates and novel drug targets were subjected to BLASTp through the NCBI protein blast server (https://blast.ncbi.nlm.nih.gov/Blast.cgi) using an E value cutoff score of 1 against the data set present in Human Microbiome Project server (https://www.hmpdacc.org/hmp/) “43021 (BioProject)” (111). The Human Microbiome Project (HMP) collected microbial strains from a disease-free, stable, 239-person adult population, which included 18 body habitats in five areas (oral, skin, nasal, gut, and urogenital), creating 5,026 microbial species compositions (112). Proteins showing a <48% similarity were identified as novel therapeutic targets and vaccine candidates and moved to the next stage. The results of this screening study ensured the chance to prevent unintended inhibition and unconscious blockage of human microflora proteins.

### Protein-protein interaction studies.

PPI analysis for selected shortlisted proteins were predicted using STRING (113). The database identifies physical (direct) and functional (indirect) interactions. PPI studies with a high confidence score (≥70%) were considered to avoid false-positive results. Inhibition of essential proteins can hamper other proteins from performing correctly. Therefore, the PPI studies of the shortlisted proteins with others of the same strain can help identify the best therapeutic targets. Proteins showing close interactions with at least three others were selected for further studies. Only an OMP with antigenicity score of >0.6 was transferred to the next stage for reverse vaccinology after homology modeling and validation. The rest were listed as probable novel drug targets.

### Homology modeling, structure optimization, and validation.

Since no exact Protein Data Bank (PDB) structure was available for the selected proteins, a BLASTp analysis was carried out in the NCBI database for each protein, where PDB proteins were chosen as the database. The templates were chosen for homology modeling considering a sequence identity of ≥85 and a query cover of ≥30. EasyModeller 4.0 software generated the best 3D structure of each selected therapeutic protein (114). The best models were subjected to ModRefiner for energy minimization and structure refinement (115). RMSD values were also calculated. Evaluation of the optimized, refined structures was carried out using the online servers PROCHECK (116) and ERRAT (117) present in the Structural Analysis and Verification Server (SAVES; https://saves.mbi.ucla.edu/) to get the stereochemical quality of the model.

### Antigenic protein selection and T-cell epitope prediction.

According to PPI analysis and antigenicity, the final vaccine target was selected among the membrane-associated proteins. The ProtParam (118) tool showed the different physicochemical parameters of the proteins. The IEDB MHC-I and MHC-II prediction tools predict MHC-I and MHC-II binding peptides, respectively (119).

### Transmembrane topology, antigenicity, population coverage, and allergenicity assessment of T-cell epitopes.

The TMHMM server predicted transmembrane helices in the protein (120), while the VaxiJen v2.0 server was used to test antigenicities of the epitopes (105). Population coverage has been evaluated for each human epitope using the IEDB analysis tool (121). Four independent servers—AllerTOP (122), AllergenFP (123), PA3P (124), and Allermatch (125)—are used to estimate epitope allergenicity for potential vaccine design.

### Toxicity and conservancy analysis.

The server ToxinPred (https://webs.iiitd.edu.in/raghava/toxinpred2/) (126) estimated the relative toxicities of top T-cell epitopes. Conservancy of the epitope has been shown to determine the degree of distribution in the homologous protein set of corresponding epitopes using the IEDB epitope conservancy analysis tool (http:/tools.iedb.org/conservancy/) (127).

### Identification of B-cell epitopes and cluster analysis.

Six different IEDB algorithms—the Kolaskar and Tongaonkar antigenicity scale (128), the Karplus and Schulz versatility prediction (129), the Chou and Fasman beta-turn prediction (130), the Emini surface accessibility prediction (131), the Parker hydrophilicity prediction (132), and the Bepipred linear epitope prediction tool (133)—were used to identify the most active B-cell epitopes in the target protein. In addition, an analysis tool for the IEDB epitope cluster was used to identify overlapping peptides among the predicted epitopes (134). Larger cassettes containing multiple epitopes were found between top epitopes of CTL, HTL, and BCL.

### Final vaccine construction.

The final vaccine was formulated using the top epitopes (T and B cells), the adjuvants, and the corresponding linkers. Three specific adjuvants, i.e., beta-defensin, ribosomal protein L7/L12, and Mycobacterium tuberculosis HABA protein (AGV15514.1), have produced three separate vaccine molecules. Adjuvants interact with TLRs and cause immune activation by polarizing CTL reactions (135). The beta-defensin adjuvants are TLR-1, -2, and -4 agonists, whereas L7/L12 ribosomal protein and HBHA protein act only as TLR-4 agonists. EAAAK, GGGS, GPGPG, and KK linkers have been used to bind the adjuvant, CTL, HTL, and B-cell epitopes, respectively. Literature reports show that linkers allow efficient *in vivo* separation of individual epitopes (136, 137). To address the problem with highly polymorphic HLAs, a PADRE sequence was also inserted into vaccine constructs.

### Allergenicity, antigenicity, physicochemical property, and secondary structure analysis of vaccine constructs.

AlgPred v.2.0 (138) sever was used to predict the nonallergic nature of the constructed vaccines. VaxiJen v2.0 server field (68) was used to suggest the superior vaccine candidate for evaluating its antigenicity. ProtParam (118) reveals the molecular weight, instability index, approximate half-life, isoelectric pH, GRAVY values, hydropathicity, aliphatic index, and other physicochemical properties of the constructs. The secondary structure of the vaccine protein was determined by PSIPRED v3.3 (139) and NetTurnP v1.0 (140).

### Vaccine tertiary structure prediction, refinement, validation, and disulfide engineering.

The RaptorX server generated a tertiary structure of the constructs based on the degree of similarity between the target protein and accessible template structure in the PDB database (141, 142). Refinement was conducted using ModRefiner (115), followed by the FG-MD refinement server (143), to improve the accuracy of the predicted 3D modeled structure. A Ramachandran plot of the refined structure was assessed by RAMPAGE (144). Disulfide bonds enhance the geometric conformation of proteins and provide significant stability. DbD2 was used to build these bonds for the vaccine designs (145). The values of chi3 selected for the residue screening were −87 to +97, while the energy value considered was <2.5.

### Protein-protein docking, molecular dynamics simulation, and interaction analysis of vaccine constructs and TLR-2.

Inflammations caused by bacterial antigen are involved with TLR-2 immune receptors present over the immune cells (5, 146, 147). The 3D structure of different MHC molecules and human TLR-2s has been obtained from the database of RCSB proteins. A molecular docking method using ClusPro (148), HDOCK (149), and PatchDock server (150) determined the binding affinities of designed vaccines with specific HLAs and TLR-2 immune receptors. The FireDock server refined the complexes generated via the PatchDock server (150). The vaccine-receptor complex’s stability was determined by contrasting the critical protein dynamics to their normal modes (151, 152). Essential dynamics is a versatile tool and an inexpensive solution to the costly atomistic simulation (151, 153).

Molecular dynamics simulation is an important step for analyzing the stability of a docked complex. Here, we evaluate the docked complex of TLR-2 and our V1 vaccine construct for stability. MD simulation was performed using the GROMACS 2020.1 package (154) using the CHARMM36 (155) force-field. The step was done to simulate the vaccine interaction with the TLR-2 in the native environment of the body. The docked complex of receptor-vaccine was solvated in a cubic box with SPC water molecules (156). To neutralize the system, 18 Cl^−^ ions were added to the system. The system underwent subsequent NVT and NPT ensemble MD simulations for 500 ps each time to maintain temperature and pressure. The system then underwent 100-ns MD simulation under equivalence at 300K and 1 bar. The simulation trajectory was then analyzed to plot the RMSD, RMSF, and Rg results. Finally, molecular interactions of the vaccine-TLR complex were analyzed using the PDBsum webserver to gain structural insights into the docked complex (157).

### Codon adaptation and *in silico* cloning.

A codon adaptation technique was used for greater expression of the vaccine protein in E. coli. The procedure was conducted by the JCAT server (158), while preventing the termination of rho’s independent transcription, the ribosome-binding prokaryote site, and the cleavage of several other restriction enzymes (i.e., HindIII and BamHI). The optimized sequence of vaccine protein V1 was reversed and then conjugated with HindIII and BamHI restriction sites at the N- and C-terminal sites, respectively. SnapGene, a restriction cloning module, was used to insert the adapted sequence between HindIII (173) and BamHI (198) of the pET28a(+) vector.
