# Supplementary material for: Proteome Exploration of Legionella pneumophila To Identify Novel Therapeutics: a Hierarchical Subtractive Genomics and Reverse Vaccinology Approach
Source: Microbiol Spectr. 2022 Jul 12;10(4):e00373-22. doi: 10.1128/spectrum.00373-22 (PMC9430848; doi:10.1128/spectrum.00373-22)
Supplement: Supplemental file 1 — Supplemental material. Download spectrum.00373-22-s0001.pdf, PDF file, 2.1 MB [file spectrum.00373-22-s0001.pdf]

Supplemental material

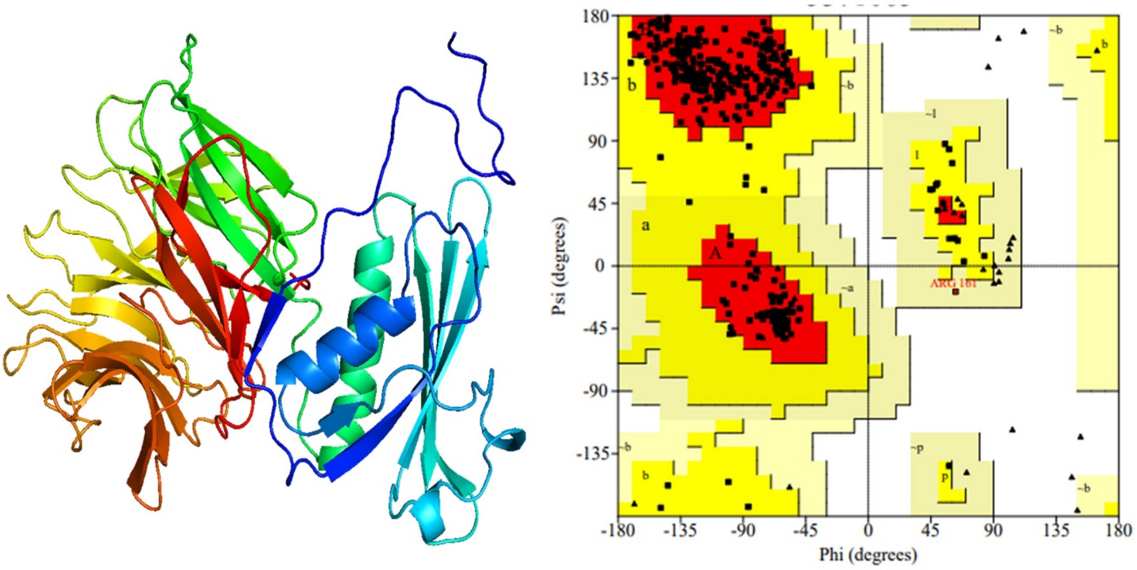

A B

Figure S1. Tertiary structure prediction of the vaccine target through EasyModeller 4.0 **(A)** and Ramachandran Plot analysis **(B)**.

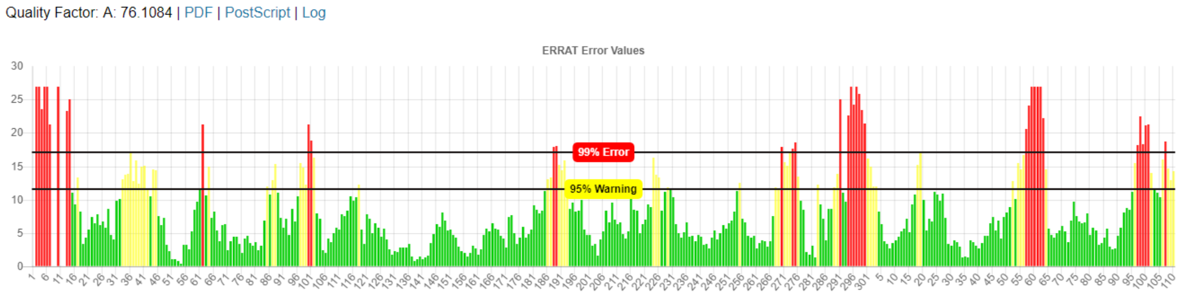

Figure S2. Quality factor analysis of modelled 3D structure of LEGPH protein TolB by ERRAT.

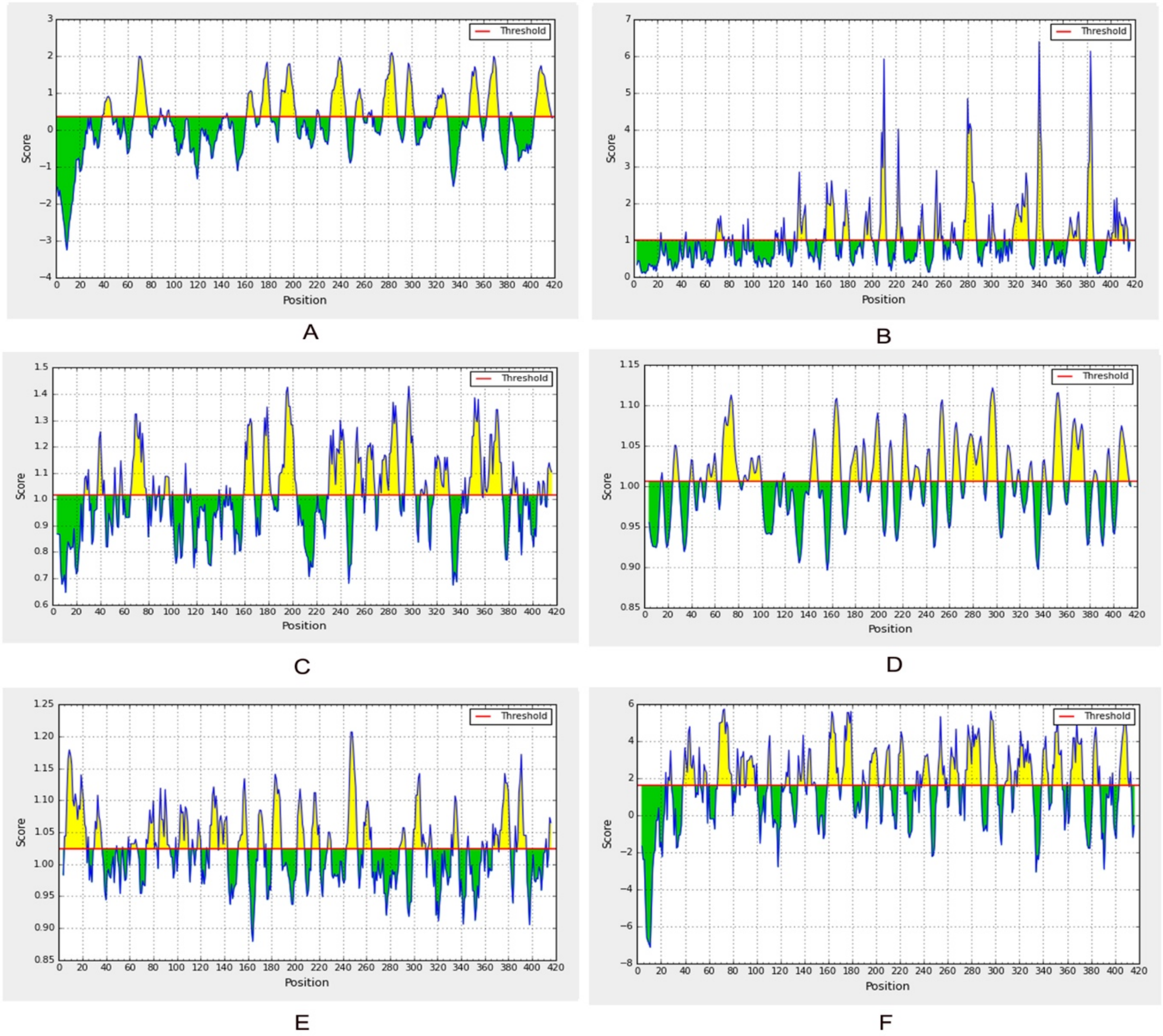

Figure S3. Prediction of B cell linear epitope and intrinsic properties for LEGPH protein Tol B using different scales (**A:** Bepipred, **B:** Surface accessibility, **C:** Emini surface, **D:** Flexibility, **E:** Antigenicity, **F:** Hydrophilicity). **(**For each graph: the x-axis and y-axis represent the position and score; residues that fall above the threshold value are shown in yellow colour; the highest peak in yellow colour identifies the most favoured position).

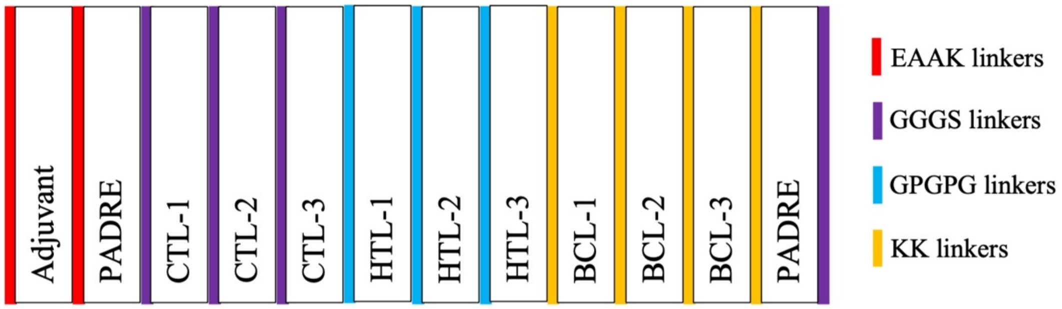

Figure S4. Schematic representation of the *L. pneumophila* vaccine constructs.

The adjuvants are attached at the N-terminal of the construct. The four linkers (EAAAK, GGGS, GPGPG and KK were used to connect the adjuvant, PADRE and epitopes together.

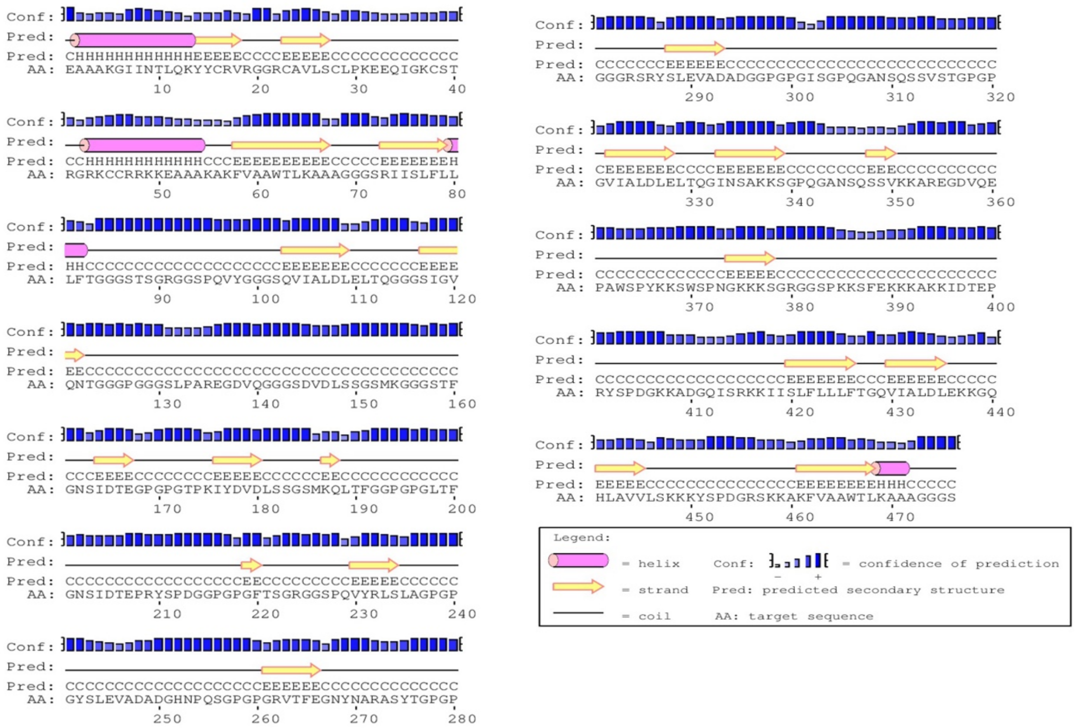

Figure S5. Secondary structure prediction of designed vaccine V1 using PESIPRED server

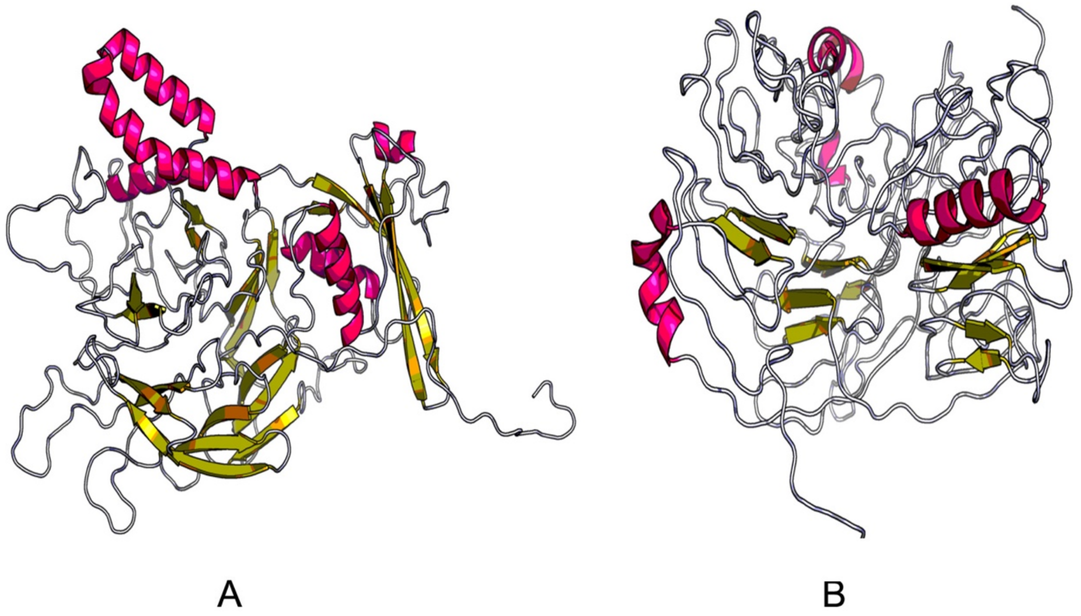

Figure S6. 3D modelled structure of vaccine protein V2 **(A)** and V3 **(B)** generated via RaptorX server.

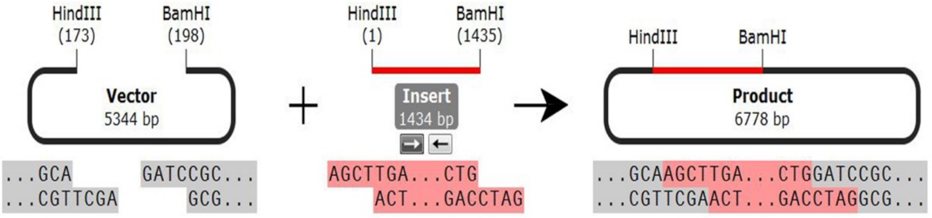

A

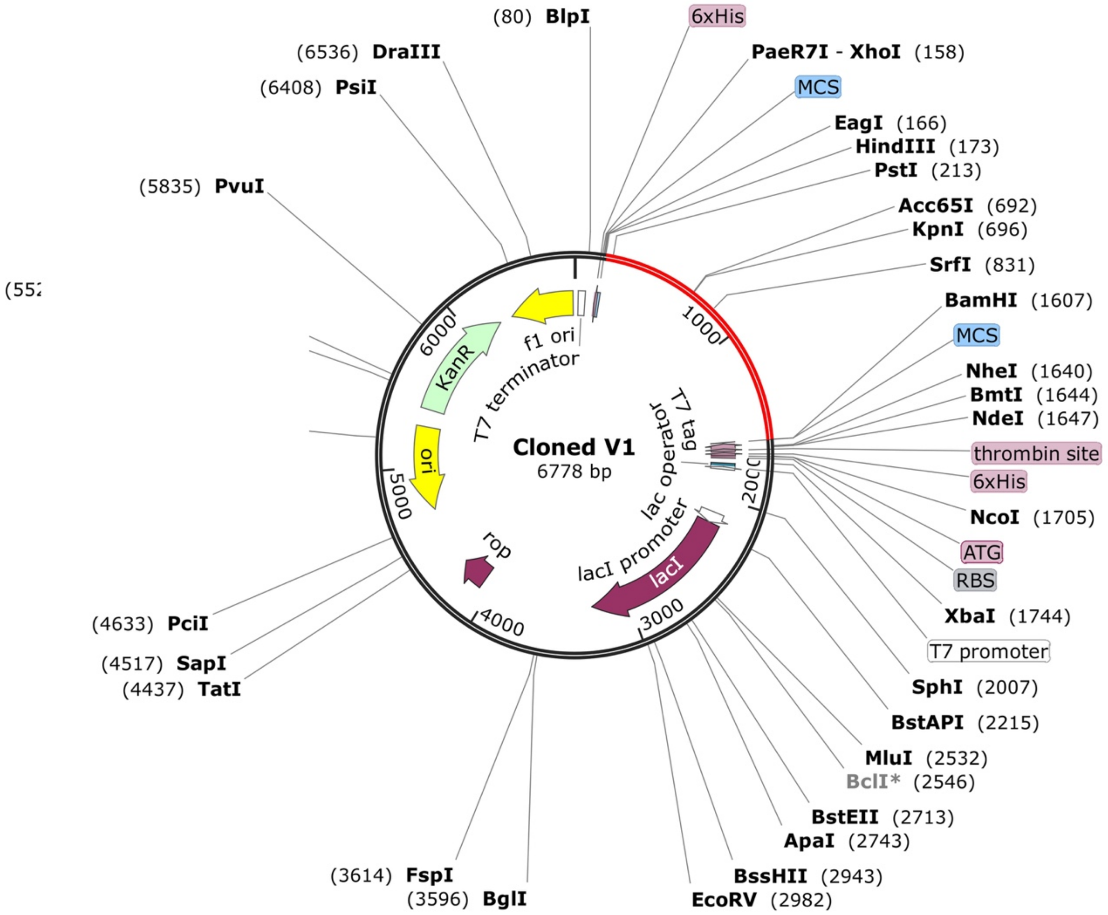

B

Figure S7. Restriction digestion **(A)** and *in silico* cloning **(B)** of the gene sequence of final vaccine construct V1 into pET28a(+) expression vector. Target sequence was inserted between HindIII (173) and BamHI (198).

Supplementary Table 1: Unique pathways of *Legionella pneumophila* retrieved from KEGG server.

Serial. no.	Pathway id	Unique Pathways
1	lpn00121	Secondary bile acid biosynthesis
2	lpn00261	Monobactam biosynthesis
3	lpn00281	Geraniol degradation
4	lpn00300	Lysine biosynthesis
5	lpn00332	Carbapenem biosynthesis
6	lpn00361	Chlorocyclohexane and chlorobenzene degradation
7	lpn00362	Benzoate degradation
8	lpn00364	Fluorobenzoate degradation
9	lpn00401	Novobiocin biosynthesis
10	lpn00460	Cyanoamino acid metabolism
11	lpn00473	D-Alanine metabolism
12	lpn00521	Streptomycin biosynthesis
13	lpn00523	Polyketide sugar unit biosynthesis
14	lpn00525	Acarbose and validamycin biosynthesis
15	lpn00540	Lipopolysaccharide biosynthesis
16	lpn00550	Peptidoglycan biosynthesis
17	lpn00623	Toluene degradation
18	lpn00625	Chloroalkane and chloroalkene degradation
19	lpn00626	Naphthalene degradation
20	lpn00627	Aminobenzoate degradation
21	lpn00643	Styrene degradation

22	lpn00680	Methane metabolism
23	lpn00903	Limonene and pinene degradation
24	lpn00930	Caprolactam degradation
25	lpn01110	Biosynthesis of secondary metabolites
26	lpn01120	Microbial metabolism in diverse environments
27	lpn01130	Biosynthesis of antibiotics
28	lpn01220	Degradation of aromatic compounds
29	lpn01501	beta-Lactam resistance
30	lpn01502	Vancomycin resistance
31	lpn01503	Cationic antimicrobial peptide (CAMP) resistance
32	lpn02020	Two-component system
33	lpn02024	Quorum sensing
34	lpn02030	Bacterial chemotaxis
35	lpn02040	Flagellar assembly
36	lpn03070	Bacterial secretion system
37	lpn00550	Peptidoglycan biosynthesis

Supplementary Table 2: KO assigned (KEGG orthlogy) proteins not involved in any metabolic pathway

Serial no.	KO assignment	Accession no.	Protein name
1	K03797	tr|Q5ZY73	Carboxy-terminal protease
2	K03294	tr|Q5ZZI4	Amino acid permease
3	K09822	sp|Q5ZYZ0	Hypothetical protein
4	K07568	sp|Q5ZU04	S-adenosylmethionine:tRNAribosyltransferase-isomerase
5	K06941	sp|Q5ZV93	Dual-specificity RNA methyltransferaseRlmN
6	K03980	tr|Q5ZS84	Probable lipid II flippaseMurJ
7	K03466	tr|Q5ZUM7	Cell division protein FtsK
8	K03089	tr|Q5ZS52	RNA polymerase sigma factor RpoH
9	K07007	tr|Q5ZS92	Hypothetical protein
10	K03656	tr|Q5ZRR4	ATP-dependent DNA helicase Rep
11	K03837	tr|Q5ZT44	Serine transporter
12	K03086	tr|Q5ZT04	LEGPH RNA polymerase sigma factor RpoD
13	K03609	tr|Q5ZUR8	Site-determining protein
14	K02600	tr|Q5ZRV3	Transcription termination/antitermination protein NusA
15	K03722	tr|Q5ZU85	ATP dependent DNA helicase
16	K03592	tr|Q5ZUM2	Peptide maturation protein PmbA
17	K19810	tr|Q5ZYS3	L-lysine 2,3-aminomutase, radical SAM domain protein
18	K05837	tr|Q5ZVR6	Peptidoglycan glycosyltransferaseMrdB
19	K03641	sp|Q5ZV69	Protein TolB
20	K03545	sp|Q5ZUD8	Trigger factor
21	K03549	sp|Q5ZW98	Probable potassium transport system protein kup 1
22	K03549	sp|Q5ZSY2	Probable potassium transport system protein kup

23	K03549	sp|Q5ZTN5	LEGPH Probable potassium transport system protein kup 2
24	K13292	sp|Q5ZRL2	Prolipoproteindiacylglyceryltransferase
25	K06915	tr|Q5ZYU2	Hypothetical protein
26	K07277	tr|Q5ZY66	Outer membrane protein assembly factor BamA
27	K09014	tr|Q5ZXX9	ABC transporter, permease
28	K02198	tr|Q5ZX71	Cytochrome c-type biogenesis protein CcmF
29	K02558	tr|Q5ZUP0	UDP-N-acetylmuramate-L-alanyl-gamma-D-glutamyl-meso-2,6-diaminoheptandioate ligase

Supplementary Table 3: Functional family classification of the hypothetical proteins by SVMProt server

**Serial. no.**	**Accession no.**	**KO assignment**	**Family name**	**Probability (%)**	**GO category**
1	sp|Q5ZYZ0	K09822	Iron-binding	98.4	GO:0005506
2	tr|Q5ZS92	K07007	Iron-binding	98.9	GO:0005506
3	tr|Q5ZYU2	K06915	All DNA-binding	85.4	GO:0003677

Supplementary Table 4: Subcellular localization of nonhomologous essential proteins involved in unique pathways of *Legionella pneumophila*

Serial. no.	Accession no.	PSORTbv3.0.2 server	CELLO v2.5 server	ngLOC server	PSLpred server	Final result
1	tr|Q5ZY73	Cytoplasmic Membrane	Outer membrane	Cytoplasmic	Cytoplasmic	Cytoplasmic
2	tr|Q5ZZI4	Cytoplasmic Membrane	Inner Membrane	Inner Membrane		Inner Membrane
3	sp|Q5ZYZ0	Cytoplasmic	Cytoplasmic	Cytoplasmic		Cytoplasmic
4	sp|Q5ZU04	Cytoplasmic	Cytoplasmic	Cytoplasmic		Cytoplasmic
5	sp|Q5ZV93	Cytoplasmic	Cytoplasmic	Cytoplasmic		Cytoplasmic
6	tr|Q5ZS84	Cytoplasmic Membrane	Inner Membrane	Inner Membrane		Inner Membrane
7	tr|Q5ZUM7	Cytoplasmic Membrane	Inner Membrane	Inner Membrane		Inner Membrane
8	tr|Q5ZS52	Cytoplasmic	Cytoplasmic	Cytoplasmic		Cytoplasmic
9	tr|Q5ZS92	Cytoplasmic	Cytoplasmic	Cytoplasmic		Cytoplasmic
10	tr|Q5ZRR4	Cytoplasmic	Cytoplasmic	Cytoplasmic		Cytoplasmic
11	tr|Q5ZT44	Cytoplasmic Membrane	Inner Membrane	Inner Membrane		Inner Membrane
12	tr|Q5ZT04	Cytoplasmic	Cytoplasmic	Cytoplasmic		Cytoplasmic

13	tr|Q5ZUR8	Multiple location	Cytoplasmic	Inner Membrane	Inner Membrane	Inner Membrane
14	tr|Q5ZRV3	Cytoplasmic	Cytoplasmic	Cytoplasmic		Cytoplasmic
15	tr|Q5ZU85	Cytoplasmic	Cytoplasmic	Cytoplasmic		Cytoplasmic
16	tr|Q5ZUM2	Cytoplasmic	Outer membrane	Cytoplasmic		Cytoplasmic
17	tr|Q5ZYS3	Cytoplasmic	Cytoplasmic	Cytoplasmic		Cytoplasmic
18	tr|Q5ZVR6	Cytoplasmic Membrane	Inner Membrane	Inner Membrane		Inner Membrane
19	sp|Q5ZV69	Periplasmic	Outer membrane	Inner Membrane	Outer membrane	Outer membrane
20	sp|Q5ZUD8	Cytoplasmic	Cytoplasmic	Cytoplasmic		Cytoplasmic
21	sp|Q5ZW98	Cytoplasmic Membrane	Inner Membrane	Inner Membrane		Inner Membrane
22	sp|Q5ZSY2	Cytoplasmic Membrane	Inner Membrane	Inner Membrane		Inner Membrane
23	sp|Q5ZTN5	Cytoplasmic Membrane	Inner Membrane	Inner Membrane		Inner Membrane
24	sp|Q5ZRL2	Cytoplasmic Membrane	Inner Membrane	Inner Membrane		Inner Membrane
25	tr|Q5ZYU2	Unknown	Inner Membrane	Cytoplasmic	Periplasmic	Multiple location

26	tr|Q5ZY66	Outer membrane	Outer membrane	Inner Membrane		Outer membrane
27	tr|Q5ZXX9	Cytoplasmic	Cytoplasmic	Cytoplasmic		Cytoplasmic
28	tr|Q5ZX71	Cytoplasmic Membrane	Inner Membrane	Inner Membrane		Inner Membrane
29	tr|Q5ZUP0	Cytoplasmic	Cytoplasmic	Cytoplasmic		Cytoplasmic
30	tr|Q5ZXL1	Cytoplasmic Membrane	Inner Membrane	Inner Membrane		Inner Membrane
31	tr|Q5ZX16	Cytoplasmic	Cytoplasmic	Cytoplasmic		Cytoplasmic
32	tr|Q5ZX17	Cytoplasmic Membrane	Inner Membrane	Cytoplasmic		Inner Membrane
33	tr|Q5ZVR5	Cytoplasmic Membrane	Outer membrane	Inner Membrane		Inner Membrane

Supplementary Table 5: Details of Protein-Protein Interactions through STRING v11.5

Target Protein	Predicted Functional Partner	Gene Name	Functions
UDP-N-acetylmuramoyl-L-alanyl-D-glutamate--2,6-diaminopimelate ligase (murE)	UDP-N-acetylmuramate--L-alanine ligase	murC	involved in the pathway peptidoglycan biosynthesis, which is part of Cell wall biogenesis
	Peptidoglycan D,D-transpeptidase FtsI	pbpB	Catalyzes cross-linking of the peptidoglycan cell wall at the division septum
	Phospho-N-acetylmuramoyl-pentapeptide-transferase	mraY	involved in the first step of the lipid cycle reactions in the biosynthesis of the cell wall peptidoglycan
	Probable peptidoglycan glycosyltransferase FtsW	ftsW	Essential cell division protein.; transports lipid-linked peptidoglycan precursors from the inner to the outer leaflet of the cytoplasmic membrane
	D-alanine--D-alanine ligase	ddl	involved in cell wall formation; catalyzes the formation of D-alanyl-D-alanine
	UDP-N-acetylglucosamine--N-acetylmuramyl-(pentapeptide) pyrophosphoryl-undecaprenol N-acetylglucosamine transferase	murG	Catalyzes the transfer of a GlcNAc subunit on undecaprenyl-pyrophosphoryl-MurNAc-pentapeptide (lipid intermediate I) to form undecaprenyl-pyrophosphoryl-MurNAc- (pentapeptide)GlcNAc (lipid intermediate II); involved in cell wall formation
	UDP-N-acetylenolpyruvoylglucosamine reductase	murB	involved in the pathway peptidoglycan biosynthesis
	UDP-N-acetylglucosamine 1-carboxyvinyltransferase	murA	involved in the pathway peptidoglycan biosynthesis; adds enolpyruvyl to UDP-N-acetylglucosamine
	UDP-N-acetylmuramoyl-tripeptide--D-alanyl-D-alanine ligase	murF	Involved in cell wall formation; catalyzes the final step in the synthesis of UDP-N-acetylmuramoyl-pentapeptide, the precursor of murein
	UDP-N-acetylmuramoylalanine--D-glutamate ligase	murD	Catalyzes the addition of glutamate to the nucleotide precursor UDP-N-acetylmuramoyl-L-alanine

			(UMA); involved in cell wall formation.
	50S ribosomal protein L15	rplO	Binds to the 23S rRNA
Trigger factor (tig)	ATP-dependent Clp protease proteolytic subunit	clpP	Cleaves peptides in various proteins in a process that requires ATP hydrolysis; role in the degradation of misfolded proteins
	60 kDa chaperonin	grol	Prevents misfolding and promotes the refolding and proper assembly of unfolded polypeptides generated under stress conditions
	Peptidoglycan D,D-transpeptidase MrdA	pbpA	Catalyzes cross-linking of the peptidoglycan cell wall
	50S ribosomal protein L2	rplB	Required for association of the 30S and 50S subunits to form the 70S ribosome, for tRNA binding and peptide bond formation
	Two component response regulator	lpg0715	Makes several contacts with the 16S rRNA in the 70S ribosome
	50S ribosomal protein L29	rpmC	-
	50S ribosomal protein L5	rplE	It contacts protein S13 of the 30S subunit (bridge B1b), connecting the 2 subunits in the 70S ribosome; Contacts the P site tRNA
	50S ribosomal protein L6	rplF	Binds to the 23S rRNA
	50S ribosomal protein L23	rplW	Binds 23S rRNA; Forms the main docking site for trigger factor binding to the ribosome
	50S ribosomal protein L15	rplO	Binds to the 23S rRNA
Probable lipid II flippase MurJ (mviN)	Biotin synthase	bioB	Catalyzes the conversion of dethiobiotin (DTB) to biotin by the insertion of a sulfur atom into dethiobiotin via a radical- based mechanism
	Leucine aminopeptidase	lpg2634	-
	Uncharacterized protein	lpg2633	-
Protein TolB (tolB)	TolA colicin import membrane protein	tolA	-
	Cell division coordinator CpoB	cpoB	Mediates coordination of peptidoglycan synthesis and outer membrane constriction during cell division

	Peptidoglycan-associated protein	pal	plays a role in outer membrane invagination during cell division; important for maintaining outer membrane integrity
	Outer-membrane lipoprotein carrier protein	lolA	Participates in the translocation of lipoproteins from the inner membrane to the outer membrane
	Tol-Pal system protein TolR	tolR	Participates in the translocation of lipoproteins from the inner membrane to the outer membrane
	Holliday junction ATP-dependent DNA helicase RuvB	ruvB	mediates the Holliday junction migration by localized denaturation and reannealing
	Tol-Pal system protein TolQ	tolQ	Participates in the translocation of lipoproteins from the inner membrane to the outer membrane
	Esterase	lpg1575	-
	Outer membrane protein, OmpA family protein	lpg0657	-
	Sodium-type flagellar protein	lpg2962	-
Probable potassium transport system protein kup 1 (kup1)	Uncharacterized protein	lpg1189	-

Supplementary Table 6: Templates used for homology modelling by EasyModeller 4.0

Protein accession no.	PDB ID	Identity (%)	Query cover
sp|Q5ZV69	4R40	41	95
	3IAX	40	99
	1C5K	40	99

Supplementary Table 7: ProtParam analysis of LEGPH protein TolB

Viral Proteins	Accession ID	Molecular Weight	Instability Index	Aliphatic Index	Theoretical pI	No. of Amino acids	Estinction Co-Efficient	Gravy
TolB_LEGPH	Q5ZV69	45387.06	38.09	89.81	8.67	419	34380	-0.204

Supplementary Table 8: Identified epitope clusters among top CTL, HTL and BCL epitopes

CTL epitope clusters	HTL epitope clusters	BCL epitope clusters
RIISLFLLLFT<br>TSGRGGSPQVY<br>QVIALDLELTQ<br>IGVQNTGGGP<br>LPAREGDVQ<br>DVDLSSGSMK<br>TFGNSIDTE	TPKIYDVDLSSGSMKQLTFG<br>LTFGNSIDTEPRYSPDG<br>FTSGRGGSPQVYRLSLA<br>YSLEVADADGHNPQS<br>RVTFEGNYNARASYT<br>GGRSRYSLEVADADG<br>ISGPQGANSQSSVST<br>VIALDLELTQGINSA	SGPQGANSQSSV<br>AREGDVQEPAWSPY<br>SWSPNGK<br>SGRGGSP<br>SFEKKKA<br>IDTEPRYSPDG<br>ADGQISR<br>IISLFLLLFTGQVIAL<br>DLE<br>GQHLAVVLSK<br>YSPDGRS

Supplementary Table 9: Protein-protein interaction after 100 ns molecular docking complex.

Receptor	Residues	Position	Bond type	Ligand	Residues	Position	Distance
Human TLR2 (Chain B)	ASN	294	Hydrogen	Vaccine (Chain D)	ILE	396	2.66
	ILE	319	Hydrogen		GLY	279	2.95
	PHE	325	Hydrogen		GLN	331	3.19
	ASP	327	Hydrogen		LYS	352	2.72
	LYS	347	Hydrogen		GLY	281	3.19
	GLU	375	Hydrogen		ARG	284	2.92
	GLU	375	Hydrogen		ARG	284	3.04
	ASP	327	Salt Bridge		LYS	352	2.72
	GLU	375	Salt Bridge		ARG	284	2.92
